# National incidence and mortality of hospitalized sepsis in China

**DOI:** 10.1186/s13054-023-04385-x

**Published:** 2023-03-04

**Authors:** Li Weng, Yang Xu, Peng Yin, Yi Wang, Yan Chen, Wei Liu, Shan Li, Jin-min Peng, Run Dong, Xiao-yun Hu, Wei Jiang, Chun-yao Wang, Pei Gao, Mai-geng Zhou, Bin Du, for the China Critical Care Clinical Trials Group (CCCCTG)

**Affiliations:** 1 Medical Intensive Care Unit, State Key Laboratory of Complex Severe and Rare Diseases, Peking Union Medical College Hospital, Peking Union Medical College and Chinese Academy of Medical Sciences, 1 Shuai Fu Yuan, Beijing, 100730 China; 2 Peking University Clinical Research Institute, Peking University First Hospital, Beijing, China; 3 Department of Medical Epidemiology and Biostatistics, Karolinska Institute, Stockholm, Sweden; 4 National Center for Chronic and Noncommunicable Disease Control and Prevention, Chinese Center for Disease Control and Prevention, 27 Nanwei Road, Beijing, 100050 China; 5 Medical Record Department, Peking Union Medical College Hospital, Chinese Academy of Medical Sciences and Peking Union Medical College, Beijing, China; 6 Department of Epidemiology and Biostatistics, School of Public Health, Peking University, Beijing, China

## Abstract

**Background:**

Sepsis is a leading cause of preventable death around the world. Population-based estimation of sepsis incidence is lacking in China. In this study, we aimed to estimate the population-based incidence and geographic variation of hospitalized sepsis in China.

**Methods:**

We retrospectively identified hospitalized sepsis from the nationwide National Data Center for Medical Service (NDCMS) and the National Mortality Surveillance System (NMSS) by ICD-10 codes for the period from 2017 to 2019. In-hospital sepsis case fatality and mortality rate were calculated to extrapolate the national incidence of hospitalized sepsis. The geographic distribution of hospitalized sepsis incidence was examined using Global Moran's Index.

**Results:**

We identified 9,455,279 patients with 10,682,625 implicit-coded sepsis admissions in NDCMS and 806,728 sepsis-related deaths in NMSS. We estimated that the annual standardized incidence of hospitalized sepsis was 328.25 (95% CI 315.41–341.09), 359.26 (95% CI 345.4–373.12) and 421.85 (95% CI 406.65–437.05) cases per 100,000 in 2017, 2018 and 2019, respectively. We observed 8.7% of the incidences occurred among neonates less than 1 year old, 11.7% among children aged 1–9 years, and 57.5% among elderly older than 65 years. Significant spatial autocorrelation for incidence of hospitalized sepsis was observed across China (Moran's Index 0.42, *p* = 0.001; 0.45, *p* = 0.001; 0.26, *p* = 0.011 for 2017, 2018, 2019, respectively). Higher number of hospital bed supply and higher disposable income per capita were significantly associated with a higher incidence of hospitalized sepsis.

**Conclusion:**

Our study showed a greater burden of sepsis hospitalizations than previous estimated. The geographical disparities suggested more efforts were needed in prevention of sepsis.

**Supplementary Information:**

The online version contains supplementary material available at 10.1186/s13054-023-04385-x.

## Background

Sepsis, defined as a life-threatening illness caused by acute infection characterized by dysregulated host inflammatory response and multiple organ dysfunction, is a leading cause of preventable death among critically ill patients [1]. With the approach of global burden of disease (GBD), the Institute for Health Metrics and Evaluation (IHME) estimated that the global incidence of sepsis declined from 60.2 million in 1990 to 48.9 million in 2017 [2]. In contrast, Fleischmann-Struzek et al. reported a 46% increase of the incidence of hospital-treated sepsis since 2008 (276 cases per 100,000 person-years) compared to the overall time period [3]. These conflicting results demonstrate the difficulty in understanding the true burden of sepsis, which is complicated by many factors, such as inconsistent and variable diagnostic criteria, lack of prospective studies, hospital-based versus population-based data, and suboptimal administrative data and hospital discharge coding [3, 4]. As a result, WHO called for robust study designs and high-quality data collection [4], especially from low- and middle-income countries (LMICs) where 85% of disease burden of sepsis resides [2–5].

By the end of 2021, China remains the most populous country in the world, with more than 1.4 billion inhabitants, accounting for 18% of the global population. Despite a few attempts to estimate the incidence of sepsis in China, available data were limited to patients admitted to intensive care unit (ICU) [6–8], highly selected and non-representative hospitals [6–8], and specific regional areas [9]. In a retrospective analysis of multiple cause of death recorded in the national mortality surveillance system (NMSS), we estimated that about 1 million sepsis-related deaths occurred in China [10]. However, population-based data on sepsis incidence are still lacking.

Furthermore, significant geographic variations in sepsis-related mortality were reported in our previous study, with 46.0% of the observed variance explained by demographics, comorbidities, and socioeconomic status [10]. However, it remains to be elucidated whether the geographic variations in sepsis-related mortality was driven by the incidence of sepsis [11, 12]. Although China has made considerable improvement over the past decades, geographic disparities still remain in healthcare availability and accessibility [13, 14]. Therefore, the impact of such geographic disparities on the incidence of sepsis warrants further exploration, especially considering the ongoing Chinese health care system reform.

In this study, we aim to estimate the national incidence of hospitalized sepsis in China and describe the geographic distribution from 2017 to 2019. In addition, we investigate the association between the incidence of hospitalized sepsis and healthcare resources.

## Methods

### Study design and data sources

This was a retrospective observational database study which complied with the Guidelines for Accurate and Transparent Health Estimates Reporting statement [15]. We used the National Data Center for Medical Service (NDCMS) and the National Mortality Surveillance System (NMSS). The project was approved by the institutional review board of Peking Union Medical College Hospital (S-K 1911). The requirement to obtain informed consents was waived because of the retrospective design.

The NDCMS is a dynamic ongoing public hospital inpatient discharge abstract database under the authority of the National Health Commission of the People's Republic of China. By the end of 2020, a total of 1923 tertiary and 2363 secondary hospitals from 31 provinces, municipalities, and autonomous regions have enrolled into NDCMS, accounting for 64.1% of all tertiary hospitals and 22.7% of all secondary hospitals in mainland China (Additional file 1: Fig. S1) [16]. All admissions in tertiary and secondary hospitals of NDCMS accounted for 45.0% of all hospital admissions in whole mainland China and 69.6% of all admissions in public hospitals during the study period [16]. NDCMS contained de-identified individual-level information of hospital admissions including patient demographics (date of birth, sex and ethnicity), hospital information (de-identified identifier, hospital type, province-level location), admission date, discharge date, diagnoses, procedures, and cost records. A unique identifier (UID) was created according to the 18-digit number of resident identity card in China for each patient. A unique global identifier (UGID) was created randomly for each hospital admission. The data qualities of NDCMS were ensured by manual review, quality control meetings, courses of staff training, and regulations for quality inspection. In our analysis, we extracted all admissions in NDCMS from 2017 to 2019 due to lack of information from secondary hospitals before 2017. After exclusion of hospitals with missing UID of ≥ 50%, a total of 3530 hospitals were included for final analysis (Additional file 1: Fig. S2).

The NMSS provided nationwide representative individual-level cause of death and mortality data covering 24.3% of the total population in mainland China (Additional file 1: Method S1) [17]. All information in the standard certificate of death in China, including age, sex, the immediate, intermediate and underlying causes of death and the place of death, was reported to the NMSS [18]. Following the WHO data quality strategies [19], the overall underreporting rate in NMSS was 12.9% [20], and inaccurate coding rate was 2.7% [17]. In our analysis, all decedents who died from 2017 to 2019 were included.

### Identification of sepsis admissions

Sepsis admissions were identified using hospital discharge diagnoses in the NDCMS database, based on the official 10-digit Chinese version of International Classification of Diseases, Tenth Revision (ICD-10) diagnosis codes (an expansion from the 4-digit WHO version [21]) and ICD-9 procedure codes [2, 22–27]. Explicit sepsis cases were those with an ICD-10 code referencing sepsis explicitly (Additional file 1: Table S1). For example, the category (3-digit) code O08 pertains to the condition “complications following ectopic and molar pregnancy”, its secondary category (4-digit) code O08.2 pertains to the specific condition “embolism following ectopic and molar pregnancy”, while its tertiary category (10-digit) code O08.200 × 006 pertains to the specific condition “embolism following abortion and ectopic and molar pregnancy (septicopyaemic)”. Therefore, O08.200 × 006 was labeled as an explicit sepsis code.

Implicit sepsis cases were identified based on the commonly used algorithm that required at least one acute infection code (Additional file 1: Table S2) and an organ dysfunction code, defined by ICD-10 diagnosis codes (Additional file 1: Table S3) or ICD-9 procedure codes (Additional file 1: Table S4). The implicit-coded algorithm was validated based on a prospective, single-center, cohort study which was designed to assess the diagnostic value of qSOFA for sepsis in general ward and ICU [28]. Among all implicit sepsis, duplicated admissions, and admissions with unreasonable age (age < 0 day or age > 120 years) or hospital length of stay (LOS, LOS < 0 day or > 365 days) information, missing sex information was excluded. Because 26–60% of children younger than 5 years old in China were not registered to resident identity [29], we used the UGID as the UID for child ≤ 6 years old with missing UID information. Thereafter, patients with missing UID or fake UID (one UID corresponds to more than 4 UGIDs per year, which means one patient has more than 4 sepsis-related admissions each year) were further excluded (Additional file 1: Fig. S2). The implicit sepsis cases included explicit sepsis codes [3].

### Identification of in-hospital sepsis-related deaths in NMSS

As per Sepsis-3 criteria [1], sepsis is clinically defined as any acute infection complicated by organ dysfunction. Based on the assumption that infections causing death were most likely sepsis, thus in the 2017–2019 death record data extracted from NMSS, sepsis-related death was identified if any ICD codes of acute infections potentially related to sepsis were listed as immediate or underlying causes of death, i.e., part I or II in the certificates of death (Additional file 1: Table S2) [10]. Sepsis-related death was further identified as in-hospital sepsis-related death if the place of death was hospital. Our previous validation study showed that this algorithm had a sensitivity of 88% and a specificity of 83% [30].

### Statistical analyses

At each calendar year from 2017 to 2019, for sepsis admissions and sepsis-related deaths identified from NDCMS and NMSS, respectively, characteristics including age, sex, and Charlson Comorbidity Index (Additional file 1: Table S5), were summarized as mean ± standard deviation (SD) or median (interquartile range, IQR) for continuous variables and as count (and proportion) for categorical variables, respectively.

### Estimation of in-hospital sepsis case fatality rate

Due to the imbalanced enrollment of tertiary and secondary hospitals in the NDCMS, at each calendar year from 2017 to 2019, 5 year age-specific (< 1, 1–4, 5–9, 10–14, 15–19, 20–24, 25–29, 30–34, 35–39, 40–44, 45–49, 50–54, 55–59, 60–64, 65–69, 70–74, 75–79, 80–84, and ≥ 85 years), sex-specific (female and male), and hospital type specific (tertiary and secondary) in-hospital sepsis case fatality rates were first estimated (point estimates were calculated as number of sepsis-related deaths occurred in hospital divided by number of all hospitalized sepsis patients at each stratum stratified by 5-year age, sex and hospital type together and CIs were estimated by binomial distribution) and then summarized to age- and sex-specific annual in-hospital sepsis case fatality rates by total probability formula in nationwide and across 31 provinces, respectively. Probability of admitted sepsis patients in tertiary or secondary hospitals at each 5 year age-specific and sex-specific stratum were represented by proportions of admitted patients in tertiary or secondary hospitals at each corresponding stratum, which were extracted from China Statistical Yearbook [16].

### Estimation of in-hospital sepsis mortality rate

The information of hospital type was not collected by NMSS, therefore, at each calendar year from 2017 to 2019, 5-year age- and sex-specific in-hospital sepsis mortality rates were directly estimated (point estimates were calculated as number of sepsis-related deaths occurred in hospital divided by number of all individuals at each stratum stratified by 5 year age and sex together and CIs were estimated by binomial distribution) in nationwide and across 31 provinces, respectively.

### Estimation of hospitalized sepsis incidence

Five-year age- and sex-specific incidences of hospitalized sepsis at each calendar year from 2017 to 2019 were calculated as 5-year age- and sex-specific in-hospital sepsis mortality rates divided by 5-year age- and sex-specific in-hospital sepsis case fatality rates in nationwide and across 31 provinces in China (CIs were estimated by delta method).

Annual standardized in-hospital sepsis case fatality rates, in-hospital sepsis mortality rates and hospitalized sepsis incidences were further calculated by using the age and sex distribution of all residents in China obtained from the 2010 China Census [31], and CIs were estimated by delta method.

Details of the above estimation process were illustrated in Additional file 1: Method S2, and the trends of varying in-hospital sepsis case fatality rates, in-hospital sepsis mortality rates and hospitalized sepsis incidences with year were tested by logistic regression on aggregate data with year as independent variable [32].

### Geographic analysis of hospitalized sepsis incidence

At each calendar year, incidence of hospitalized sepsis across 31 provinces in China were first examined by Global Moran's Index to determine whether the spatial distribution of incidence is owing to chance. When spatial randomness was rejected by spatial autocorrelation test based on Global Moran's Index with *p* value < 0.05, Local Moran's Index were further calculated to identify statistically significant spatial clusters (high-high cluster, low-low cluster, high-low cluster and low–high cluster). Further explanations of Global and Local Moran's Index were detailed in Additional file 1: Method S3.

To further explore the influence of healthcare resources on the hospitalized sepsis incidence at provincial level, a spatial autocorrelation model was fitted to test the association between the hospitalized sepsis incidence and the number of physicians, the number of nurses, hospital bed supply per 10,000 population and disposable income per capita at each province [16]. We excluded Shanghai and Beijing from analysis because non-local residents accounted for 40.12% and 37.21% of the hospitalizations in Shanghai and Beijing, respectively [33].

### Sensitivity analysis

To ensure that the estimation of incidence was not affected by inclusion or exclusion of hospitals from NDCMS at each calendar year, we re-estimated in-hospital sepsis case fatality rates only using data from 3268 hospitals which were included in NDCMS continuously from 2017 to 2019, and recalculated hospitalized sepsis incidences.

Analyses were conducted in R software, version 3.4.3.

## Results

### Study population

#### Characteristics of hospitalized sepsis in NDCMS

From 2017 to 2019, we identified 9,455,279 patients with 10,682,625 sepsis admissions, including 2,393,848 patients with 2,564,750 admissions as explicit sepsis. Number of sepsis admissions increased from 2,715,999 admissions from 3315 hospitals in 2017, 3,444,660 admissions from 3403 hospitals in 2018, to 4,521,966 admissions from 3496 hospitals in 2019. The mean age of sepsis patients increased steadily from 47 ± 32 years in 2017 to 51 ± 30 years in 2019, among whom 59.6% were male. Comorbidities were common and increased from 71% in 2017 to 75.1% in 2019 (Table 1). The most common comorbidities in 2019 were congestive heart failure (24.4%), cerebrovascular disease (20.3%), and chronic pulmonary disease (20.1%). Similar findings were observed in patients with explicit sepsis (Additional file 1: Table S6). A total of 7,863,805 (73.6%) sepsis admissions occurred in tertiary hospitals during the study period. The admissions in each province across China were shown in Additional file 1: Table S7. Respiratory failure was the most common organ dysfunctions among sepsis patients, while 23.0% of patients had 2 or more organ dysfunctions. The distributions of admissions for implicit sepsis and explicit sepsis in tertiary and secondary hospitals and across 31 provinces were similar. (Additional file 1: Table S8).

**Table 1:** Demographic and Clinical Characteristics of Implicit-Coded Sepsis in NDCMS and NMSS from 2017 to 2019

	2017	2018	2019
Sepsis-related admission†			
Number of hospitals	3315	3403	3496
Number of admissions	2,715,999	3,444,660	4,521,966
Number of patients	2,452,915	3,052,599	3,949,765
Characteristics			
Age, years (SD)	47 ± 32	50 ± 31	51 ± 30
Female, *n* (%)	991,021 (40.4)	1,231,287 (40.6)	1,601,354 (40.6)
Charlson comorbidity index			
None	711,393 (29.0)	821,534 (27.1)	981,801 (24.9)
1	541,863 (22.2)	661,597 (21.9)	821,313 (20.9)
2–4	891,741 (36.3)	1,141,125 (37.6)	1,541,997 (39.1)
> 4	301,918 (12.3)	401,333 (13.4)	591,654 (15.2)
Comorbidities, *n* (%)			
Myocardial infarction	71,013 (2.9)	91,497 (3.0)	121,208 (3.1)
Congestive heart failure	571,766 (23.4)	731,272 (24.0)	961,826 (24.4)
Peripheral vascular disease	121,240 (5.1)	171,165 (5.8)	261,608 (6.8)
Cerebrovascular disease	421,972 (17.5)	571,132 (18.7)	801,033 (20.3)
Dementia	21,624 (1.1)	31,814 (1.2)	51,799 (1.3)
Chronic pulmonary disease	461,668 (18.9)	611,075 (20.1)	831,529 (21.3)
Rheumatic disease	21,802 (1.2)	31,650 (1.2)	41,067 (1.2)
Peptic ulcer disease	51,474 (2.4)	71,071 (2.6)	111,991 (2.8)
Mild liver disease	411,055 (16.8)	521,320 (17.1)	721,643 (18.3)
Moderate or severe liver disease	271,767 (11.3)	381,856 (12.5)	531,629 (13.5)
Diabetes without chronic complication	311,793 (12.7)	401,764 (13.2)	551,549 (13.9)
Diabetes with chronic complication	71,935 (3.1)	101,431 (3.3)	141,791 (3.6)
Hemiplegia or paraplegia	1,159 (0.3)	11,277 (0.4)	21,005 (0.5)
Renal disease	311,686 (12.9)	361,735 (11.9)	511,651 (13.1)
Malignancy	221,250 (9.0)	291,540 (9.7)	411,802 (10.5)
Metastatic solid tumor	61,773 (2.8)	91,557 (3.2)	141,979 (3.6)
AIDS/HIV	11,777 (0.6)	11,518 (0.6)	21,766 (0.6)
Sepsis-related death‡			
Numbers of decedents	262,935	271,095	272,698
Age, years (SD)	74 (18)	75 (18)	75 (18)
Female, *n* (%)	109,613 (41.7)	112,268 (41.4)	112,294 (41.2)
Comorbidities, *n* (%)			
Chronic pulmonary disease	139,623 (53.1)	139,622 (51.5)	134,258 (49.2)
Malignancy	21,735 (8.3)	21,986 (8.1)	22,505 (8.3)
Cerebrovascular disease	17,017 (6.5)	19,271 (7.1)	21,506 (7.9)
Cardiovascular diseases	6099 (2.3)	6654 (2.5)	6894 (2.5)
Diabetes	2520 (1)	2736 (1)	3044 (1.1)
Renal disease	593 (0.2)	710 (0.3)	673 (0.2)
Place of death, *n* (%)			
Hospital	70,881 (27.0)	76,066 (28.1)	81,905 (30.0)
Home	183,389 (69.7)	187,421 (69.1)	182,791 (67.0)

*NDCMS* National Data Center for Medical Service; *NMSS* National Mortality Surveillance System; *COPD* Chronic obstructive pulmonary disease; *Malignancy* Any malignancy, including lymphoma and leukemia, except malignant neoplasm of skin; *HIV* Human immunodeficiency virus; *SD* Standard deviation

†: Identified from NDCMS

‡: Identified from NMSS

#### Characteristics of sepsis-related deaths in NMSS

Among 6,178,318 deaths occurred from 2017 to 2019, a total of 806,728 (13.1%) were sepsis-related deaths. Among sepsis-related deaths, 228,852 (28.4%) occurred during hospitalization, while the proportion of in-hospital death continuously increased from 27.0% in 2017 to 30.0% in 2019 (Table 1). Almost 60% of sepsis-related deaths were male, and chronic pulmonary disease was the most common comorbidity (53.1%, 51.5% and 49.2% in 2017–2019 respectively).

### Incidence of hospitalized sepsis

We estimated that a total of 4,781,280 (95% CI 4,591,740–4,970,821), 5,040,777 (95% CI 4,846,307–5,235,247), 6,050,532 (95% CI 5,830,876–6,270,187) cases of hospitalized sepsis occurred across China in 2017, 2018 and 2019, respectively. Overall, annual nationwide age-standardized incidence of hospitalized sepsis for both male and female markedly increased from 328.25 (95% CI 315.41–341.09) cases per 100,000 in 2017 to 421.85 (95% CI 406.65–437.05) in 2019 (Table 2). The corresponding standardized in-hospital sepsis case fatality rate gradually fell from 5.06% (95% CI 5.00–5.13%) in 2017 to 4.46% (95% CI 4.42–4.50%) in 2019. On the contrary, the corresponding standardized in-hospital sepsis mortality rate gradually increased from 17.32 (95% CI 16.67–17.97) per 100,000 population in 2017 to 19.16 (95% CI 18.49–19.83) in 2019. Similar results were observed across 31 provinces in China. (Additional file 1: Tables S9 and S10).

**Table 2:** Incidence, Case Fatality Rate and Mortality Rate of Implicit-coded Sepsis from 2017 to 2019

	2017	2018	2019
*Crude*			
Total			
Annual sepsis incidence, per 100,000 population	308.70 (305.95–311.45)	332.50 (329.72–335.28)	370.53 (367.61–373.45)
In-hospital case fatality rate, %	6.83 (6.80–6.86)	6.78 (6.75–6.81)	6.53 (6.51–6.56)
In-hospital mortality rate, per 100,000 population	21.08 (20.92–21.24)	22.56 (22.4–22.72)	24.21 (24.04–24.38)
Female			
Annual sepsis incidence, per 100,000 population	262.75 (258.89–266.61)	283.79 (279.85–287.73)	320.15 (315.97–324.33)
In-hospital case fatality rate, %	6.07 (6.02–6.12)	5.95 (5.90–5.99)	5.68 (5.64–5.71)
In-hospital mortality rate, per 100,000 population	15.96 (15.77–16.15)	16.87 (16.67–17.07)	18.17 (17.96–18.38)
Male			
Annual sepsis incidence, per 100,000 population	353.92 (349.96–357.88)	380.64 (376.65–384.63)	421.29 (417.13–425.45)
In-hospital case fatality rate, %	7.35 (7.30–7.39)	7.36 (7.32–7.40)	7.12 (7.09–7.16)
In-hospital mortality rate, per 100,000 population	26.00 (25.76–26.24)	28.02 (27.77–28.27)	30.01 (29.75–30.27)
*Standardized*†			
Total			
Annual sepsis incidence, per 100,000 population	342.06 (328.5–355.62)	359.26 (345.4–373.12)	429.71 (414.11–445.31)
In-hospital case fatality rate, %	5.06 (5.00–5.13)	4.88 (4.83–4.94)	4.46 (4.42–4.50)
In-hospital mortality rate, per 100,000 population	17.32 (16.67–17.97)	17.55 (16.90–18.20)	19.16 (18.49–19.83)
Female			
Annual sepsis incidence, per 100,000 population	269.12 (255.55–282.69)	275.72 (262.16–289,28)	343.37 (327.75–358.99)
In-hospital case fatality rate, %	4.36 (4.26–4.45)	4.24 (4.16–4.32)	3.77 (3.71–3.84)
In-hospital mortality rate, per 100,000 population	11.72 (11.19–12.25)	11.70 (11.17–12.23)	12.95 (12.41–13.49)
Male			
Annual sepsis incidence, per 100,000 population	418.54 (403.49–433.59)	445.89 (430.5–461.28)	519.06 (502.14–535.98)
In-hospital case fatality rate, %	5.76 (5.67–5.85)	5.52 (5.45–5.59)	5.14 (5.08–5.20)
In-hospital mortality rate, per 100,000 population	24.11 (23.32–24.90)	24.61 (23.83–25.39)	26.66 (25.85–27.47)

Data in parentheses are 95% confidence intervals. In-hospital sepsis case fatality rate = the proportion of patients with sepsis who die in hospitals among all hospitalized sepsis patients over one calendar year; In-hospital sepsis mortality rate = the proportion of all in-hospital sepsis-related deaths among the whole population over one calendar year

†: Refers to age standardized for sex

**Fig. 1:**
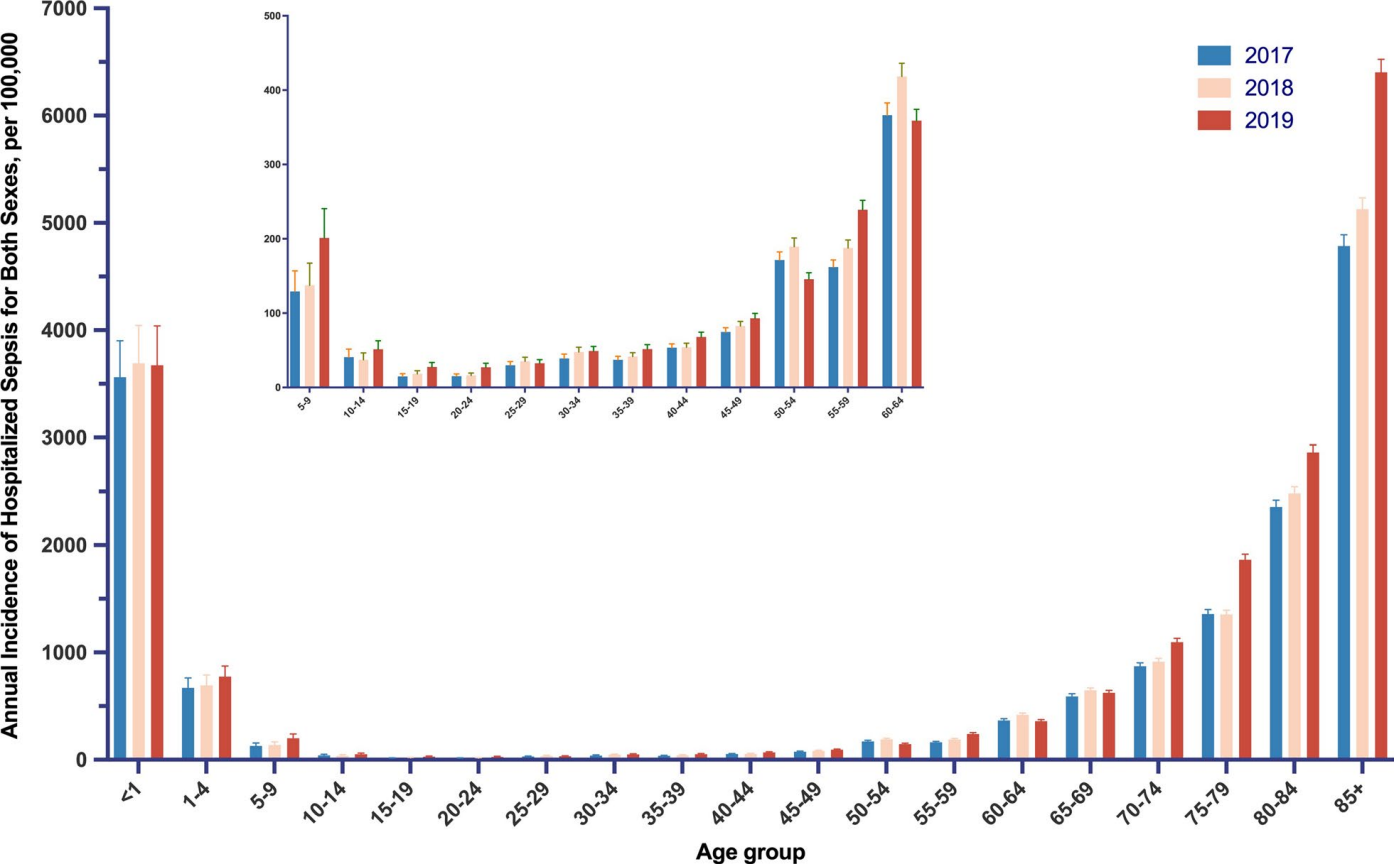
Age-specific Annual Incidence of Hospitalization with Sepsis per 100,000 Population for Both Sexes in China from 2017 to 2019. The error bars are 95% confidence intervals

The age-standardized incidence of hospitalized sepsis was higher for male than female during the study period [e.g., 519.06 (95% CI 502.14–535.98) per 100,000 vs. 343.37 (95% CI 327.75–358.99) per 100,000, in 2019]. We found the incidence of hospitalized sepsis was high at age < 1 year, decreased swiftly after ages 9 years, increased steadily throughout most of adulthood, followed by a steep increase after ages 60 years, and peaked after ages > 85 years (Fig. 1). During the 3-year study period, 8.7% of the incidences occurred among neonates less 1 year old, 9.2% among children aged 1–4 years, 2.5% among children aged 5–9 years, 19.6% among early elderly aged 65–74 years, and 37.9% among late elderly aged more than 75 years.

### Geographic patterns of hospitalized sepsis incidence

**Fig. 2:**
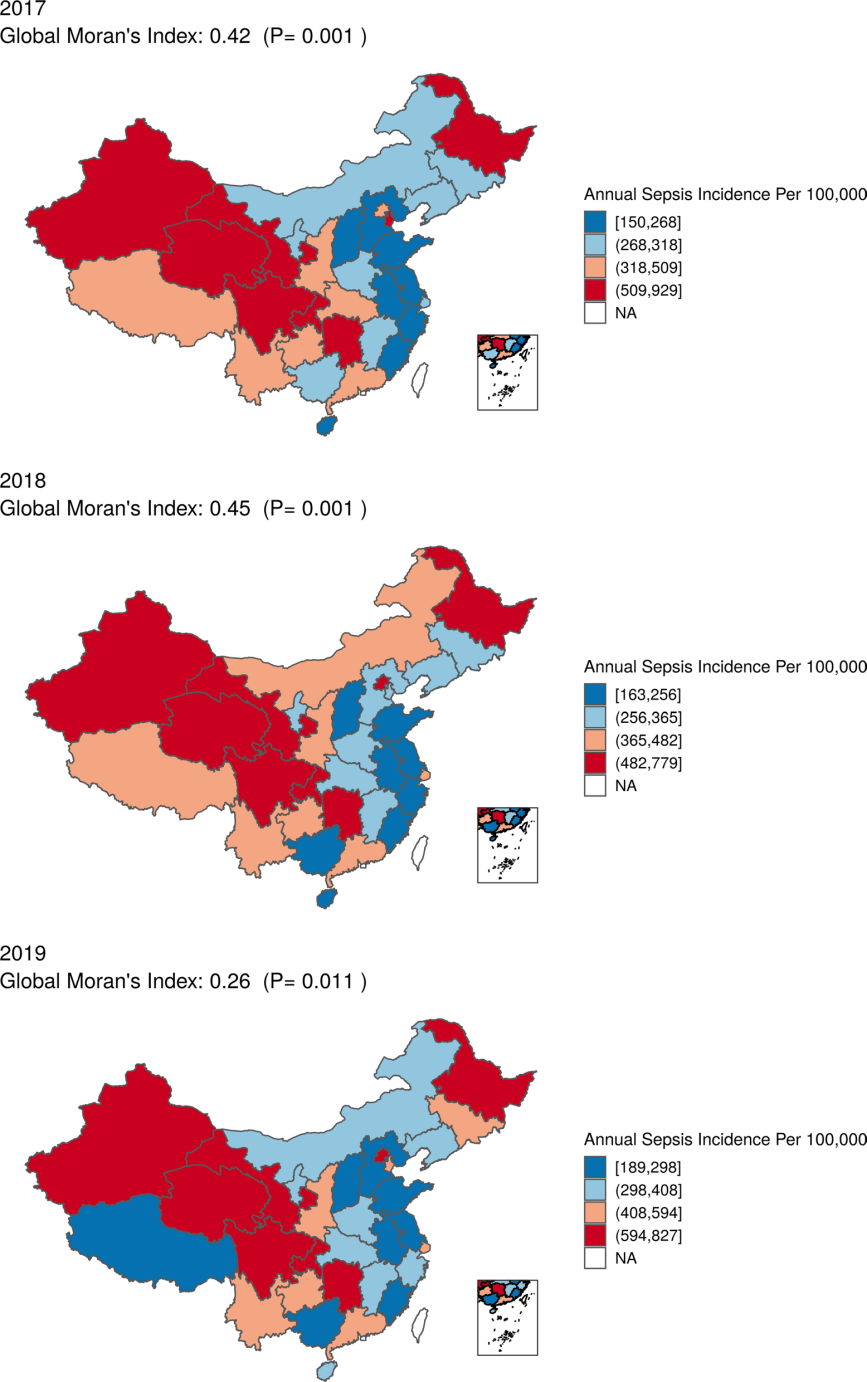
Province-specific standardized annual incidence of sepsis hospitalization per 100,000 population in China from 2017 to 2019. The estimate of incidence was added to each color-based category with a quantile classification, e.g., dark blue means an incidence between minimum and 25% quantile

**Fig. 3:**
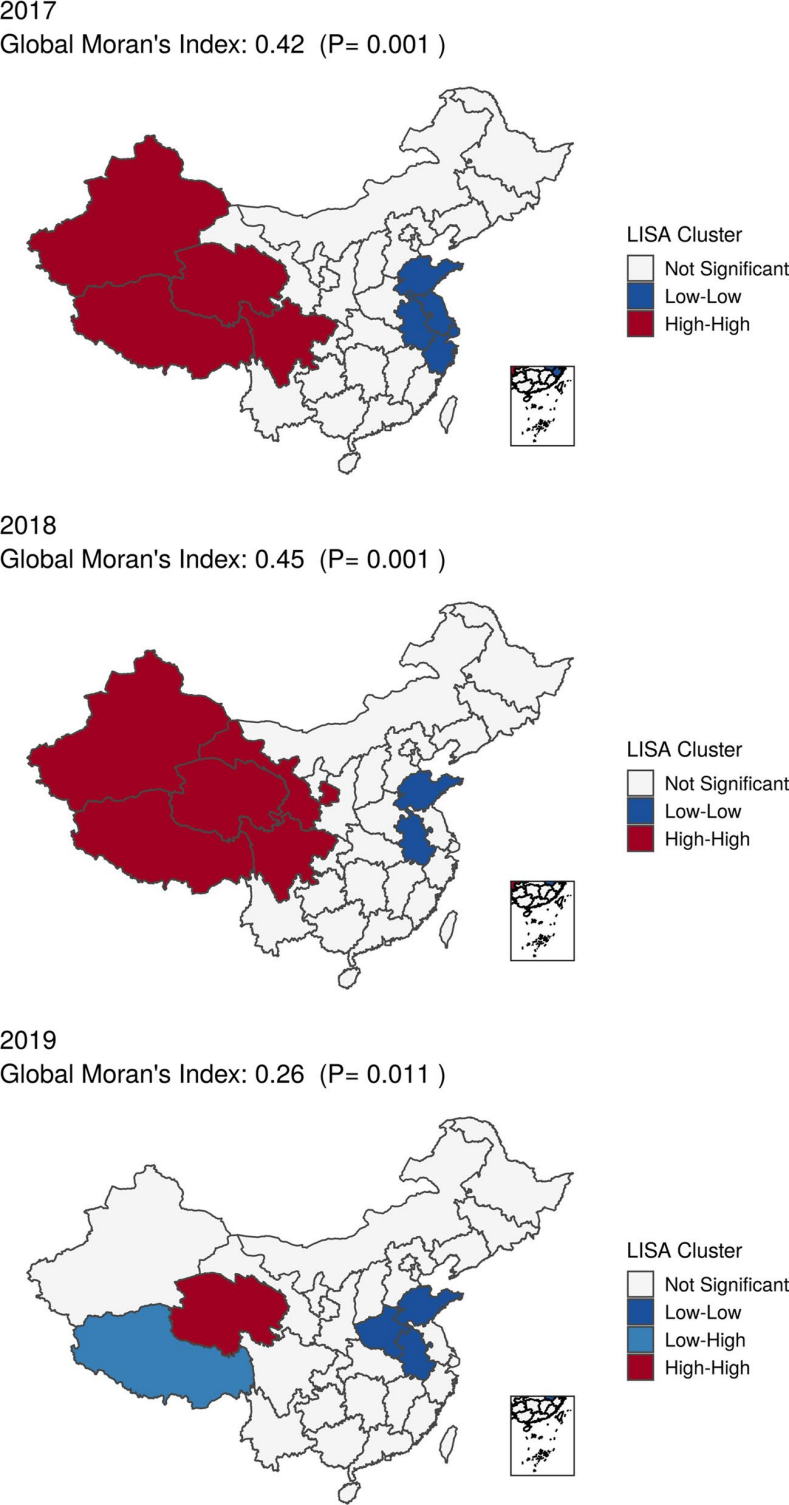
Geographic Autocorrelation of Annual Incidence of Hospitalization with Sepsis in China from 2017 to 2019

Substantial geographic variations for incidence of hospitalized sepsis were observed across China and confirmed by spatial autocorrelation test based on global Moran's Index with *p* values all < 0.05 from 2017 to 2019 (Fig. 2). Spatial autocorrelation analysis based on local Moran's Index showed that the provinces with high incidence of hospitalized sepsis were concentrated in the north-western China, while the incidence was low along the contiguous provinces in the south-eastern China (Fig. 3).

Results from spatial autocorrelation model found that higher number of hospital bed supply per 10,000 population and higher disposable income per capita were significantly associated with a higher incidence of hospitalized sepsis, while higher number of physicians per 10,000 population had a contrast influence on the incidence of hospitalized sepsis. (Additional file 1: Table S11).

### Sensitivity analysis

Similar to the primary analysis, our sensitivity analysis showed that the standardized annual incidence of hospitalized sepsis per 100,000 population was 328.12 (95% CI 315.29–340.95) in 2017, 350.09 (95% CI 336.70–363.48) in 2018, and 422.38 (95% CI 407.15–437.61) in 2019, respectively (Additional file 1: Table S12).

### Validation of the implicit-coded strategy

A total of 647 patients were enrolled into the validation cohort during the 6 month study period. Sepsis was confirmed in 103 (75%) ICU patients and 229 (56%) non-ICU patients according to the Third International Consensus Definitions for Sepsis defined as a change in SOFA score ≥ 2 points consequent to an infection. Overall, the implicit-coded strategy provided a positive predictive value (PPV) of 85.40% (95% CI 80.53–89.25%) for both ICU and non-ICU patients (Additional file 1: Table S13).

## Discussion

In this nationwide population-based study, we estimated that 4.8 million to 6.1 million hospitalized sepsis occurred annually in China from 2017 to 2019. More than 20.0% of the incidences we observed occurred among children under 10 years of age, and 57.5% of cases occurred among elderly individuals older than 65 years of age. In addition, we found significant geographic clustering of hospitalized sepsis throughout China. These findings provided important evidence to gain a true insight into the burden of sepsis in LMICs.

Our estimated incidence of 342.06 (95% CI 328.50–355.62) cases per 100,000 population in 2017 was similar to that of in Western Pacific Region [352.10 (95% CI 178.32–695.24) cases per 100,000 population], and lower than that in Pan-American Region [415.139 (95% CI 238.423–722.836) cases per 100,000 population], according to the systematic review of WHO [3]. However, the incidence of hospitalized sepsis in 2017 in our study was higher than the estimations of IHME [214.8 (95% CI 171.6–274.4) cases per 100,000 population] [2], although comparable to that in Southern Latin America [359.4 (95% CI 288.1–455.4) cases per 100,000 population]. These conflicting findings were probably attributable to the different ICD identification algorithms for implicit sepsis. Based on our previous validation of sepsis-related death [10], we did not require infection codes as the underlying cause to avoid underestimation. In addition, we extrapolated incidence from in-hospital, instead of in-and out-hospital, sepsis case fatality and mortality rate. As we previously reported, less than 30% of sepsis-related deaths occurred in hospitals annually during the study period [10, 34]. Without considering the place of death, the estimates would inevitably be biased. Another possible reason was the non-representative vital registration data of one province in China (Taiwan) used in the IHME's study. In comparison, we estimated sepsis-related deaths from the national representative dataset of NMSS and sepsis case fatality rate from a cohort with over 10 million admissions across 31 provinces in China. The representative dataset might also explain the higher incidence and lower in-hospital case fatality rate than those in our previous study, which were estimated based on an older population (mean age, 77 vs. 50 years) with a higher proportion of comorbidity (90% vs. 27%), and a developed subdistrict area in Beijing with adequate health care resources [9, 35].

The increased trend in hospitalized sepsis in our study was consistent with previous systematic review [3, 4], despite differences in the methods used. We also observed a decreased in-hospital case fatality rate and an increased in-hospital mortality rate. Meanwhile, there is tremendous variability in the incidence of sepsis across provinces (Fig. 2, Additional file 1: Table S10). There are several possible explanations for these findings. First, improved healthcare quality and coverage in the past decades ensured greater access to healthcare services for the whole population in China [14]. It was estimated that the hospital admission rate increased by 1% per annum, representing an absolute number of 10 million admissions [16]. This enhanced healthcare delivery system might significantly contribute to the observed increased cases of hospitalized sepsis. Second, the growing prevalence of non-communicable disease (NCD) in China might have a substantial impact on the burden of sepsis. According to the subnational GBD analysis, NCDs had become the leading causes of deaths in China [36]. A number of NCDs, including diabetes, cardiovascular diseases, malignancies, and rheumatological diseases, were associated with increased risk of infection death [37]. This causal link between NCDs and infectious diseases might play an important role in the subsequent emergence of sepsis. As shown in our study, most of the implicit sepsis were complicated by NCDs. Third, the China's population was rapidly aging. The age-related immune dysfunction reduced physiologic reserve capacity, and chronic underlying diseases of the elderly individuals were associated with susceptibility to infections [4]. It should be noted that an increasing trend was observed in most of the 5 year age group throughout the study period, suggesting the aging might only partially account for the trend. Fourth, there was a marked increased phenomenon of going home to die (GHTD), i.e., decreased proportion of deaths occurred in-hospital, according to our previous study based on a long-term follow-up cohort with over 0.5 million adults [34]. Indeed, the decreased proportion of deaths occurred in-hospital and the increased absolute number of in-hospital deaths was in line with the reverse trend of case fatality and mortality rates. Fifth, the improvements in management of sepsis might contribute to decreased case fatality. The short study period of 3 years supports the view that the accessibility and availability of ICU resources rather than improvement of clinical practice might partially explain our findings. Sixth, it was unclear whether incidence and trends of sepsis derived from electronic health record based clinical data were different from ICD-based estimates, similar to the findings of Rhee and his colleague [27]. Further investigation was needed to understand the reason for the changes.

Population-based studies of sepsis epidemiology in LMICs remained scarce. In the present study, we attempted to provide a comprehensive assessment of the incidence of hospitalized sepsis in China, a middle-income country with 1/5 of the world population. Our findings reinforced the evidence on the burden of sepsis worldwide [4], in LMICs in particular. The current definition of sepsis made it inapplicable to collect high-quality clinical and laboratory data for sepsis surveillance at national level in resource-limited areas. On the other hand, the scattered data in resource-limited countries remained a challenge to generate a national representative dataset. By reconciling unlinked independent administrative data sources, the codes-based surveillance strategy in our study offered a feasible low-cost approach to record the burden of sepsis in LMICs when available.

We observed significant geographic clustering of hospitalized sepsis and in-hospital mortality rates across China. Contrary to the Sepsis Belt in USA [11, 12], we found higher incidence and mortality were related to higher disposable income per capita. Increased hospitalizations accompanied by increased in-hospital deaths in resource-rich area might account for this association. The imbalanced relationship between rapid economic growth in China and lagging development of healthcare delivery system, e.g., healthcare professionals, might explain the inconsistent findings and the change in clusters during the study period as shown in Fig. 3. Our findings contribute additional evidence from LMICs regarding the influence of healthcare resources and socioeconomic status on sepsis prevention. Moreover, the unanticipated association between higher accessibility to hospital beds and higher incidence highlighted the importance of high-quality healthcare services in reducing sepsis burden. Therefore, the improvement in supply of healthcare professionals was paramount to bridge the healthcare resource gaps and address the quality of care in resource-limited areas.

Our study was subject to several limitations. First, this was an ICD-based study for sepsis surveillance. Numerous reports indicated that studies relying exclusively on administrative data had the risk to underestimate or overestimate the true burden of sepsis depending on the ICD code strategy. However, in LMICs, considering the requirement of nationally representative and individual-level data sources, ICD-based approach might be the only feasible way to extrapolate burden of sepsis. Second, the non-random sampling of hospitals in the NDCMS might introduce bias into the estimation of case fatalities. To our knowledge, the random sampling of nationally representative hospitals had not been developed. Over 10 million hospitalized sepsis were identified in our study. The large sample size might ensure the representative, avoiding selection bias. Third, increased recognition of sepsis by clinicians might have an impact on our estimation. However, the sepsis case fatality decreased by 0.6% with an increased incidence of 25.6% during the study period. The clinical documentation practice could only partially account for our findings. Fourth, only sepsis and sepsis-related deaths occurred in hospitals were enrolled in our study. Further investigation was warranted for a comprehensive estimation of sepsis burden in China.

Our study provides evidence that although in-hospital sepsis case fatality has steadily decreased in China, the hospitalized sepsis and in-hospital mortality increased. Our findings suggested that the true burden of sepsis remained a challenge to health care system in LMICs. More effects were needed to enhance the delivery of health care professionals except for the expansion of hospital beds.

## Conclusion

Our study showed a greater burden of sepsis hospitalizations than previous estimated. The geographical disparities suggested more efforts were needed in prevention of sepsis.

## Supplementary Information

**Additional file 1: Fig. S1.** Percentage of hospitals enrolled in NDCMS by province from 2017 to 2019 † ‡. **Fig. S2.** Enrollment of Admissions in NDCMS. **Method S1.** Description of Sampling Strategy and Death Data Collection in the National Mortality Surveillance System (NMSS). **Table S1.** Explicit ICD-10-CM codes of sepsis. **Table S2.** ICD-9-CM and ICD-10-CM Codes for Identification of Infection in NDCMS and Sepsis-related Death in NMSS. **Table S3.** ICD-10-CM codes of organ dysfunction. **Table S4.** ICD-9-CM codes of organ dysfunction related procedures. **Table S5.** ICD-10-CM codes of Charlson Comorbidity Index. **Method S2.** Estimation of in-hospital sepsis case fatality rate, in-hospital sepsis mortality rate and hospitalized sepsis incidence. **Method S3.** Explanations of Global and Local Moran's Index. **Table S6.** Demographic and Clinical Characteristics of Patients with Explicit-coded Sepsis in NDCMS from 2017 to 2019. **Table S7.** Characteristics of Admissions for Implicit-Coded Sepsis From 2017 to 2019. **Table S8.** Characteristics of Admissions for Explicit-Coded Sepsis From 2017 to 2019. **Table S9.** Crude Province-specific Incidence, Case Fatality Rate and Mortality Rate of Implicit-coded Sepsis from 2017 to 2019. **Table S10.** Standardized Province-specific Incidence, Case Fatality Rate and Mortality Rate of Implicit-coded Sepsis from 2017 to 2019. **Table S11.** Association between Incidence of Implicit-coded Sepsis Hospitalization and Healthcare Resources Availability from 2017 to 2019. **Table S12.** Sensitivity Analysis of Incidence, Case Fatality Rate and Mortality Rate of Implicit-coded Sepsis for Both Sexes from 2017 to 2019. **Table S13.** Validation of Implicit-Coded Strategy for Identification of Sepsis in ICU and Non-ICU Settings abc.

## Data Availability

All data generated or analyzed during this study are included in this published article.

## Abbreviations

GBD: Global burden of disease
IHME: Institute for Health Metrics and Evaluation
LMICs: Low- and middle-income countries
ICU: Intensive care unit
NMSS: National mortality surveillance system
NDCMS: National Data Center for Medical Service
UID: Unique identifier
UGID: Unique global identifier
ICD-10: International classification of diseases, tenth revision
IQR: Interquartile range
CI: Confidence intervals
NCD: Non-communicable disease
