# Supplementary material for: National incidence and mortality of hospitalized sepsis in China
Source: Crit Care. 2023 Mar 4;27:84. doi: 10.1186/s13054-023-04385-x (PMC9985297; doi:10.1186/s13054-023-04385-x)
Supplement: Supplementary file 1 — Additional file 1: Fig. S1. Percentage of hospitals enrolled in NDCMS by province from 2017 to 2019 † ‡. Fig. S2. Enrollment of Admissions in NDCMS. Method S1. Description of Sampling Strategy and Death Data Collection in the National Mortality Surveillance System (NMSS). Table S1. Explicit ICD-10-CM codes of sepsis. Table S2. ICD-9-CM and ICD-10-CM Codes for Identification of Infection in NDCMS and Sepsis-related Death in NMSS. Table S3. ICD-10-CM codes of organ dysfunction. Table S4. ICD-9-CM codes of organ dysfunction related procedures. Table S5. ICD-10-CM codes of Charlson Comorbidity Index. Method S2. Estimation of in-hospital sepsis case fatality rate, in-hospital sepsis mortality rate and hospitalized sepsis incidence. Method S3. Explanations of Global and Local Moran's Index. Table S6. Demographic and Clinical Characteristics of Patients with Explicit-coded Sepsis in NDCMS from 2017 to 2019. Table S7. Characteristics of Admissions for Implicit-Coded Sepsis From 2017 to 2019. Table S8. Characteristics of Admissions for Explicit-Coded Sepsis From 2017 to 2019. Table S9. Crude Province-specific Incidence, Case Fatality Rate and Mortality Rate of Implicit-coded Sepsis from 2017 to 2019. Table S10. Standardized Province-specific Incidence, Case Fatality Rate and Mortality Rate of Implicit-coded Sepsis from 2017 to 2019. Table S11. Association between Incidence of Implicit-coded Sepsis Hospitalization and Healthcare Resources Availability from 2017 to 2019. Table S12. Sensitivity Analysis of Incidence, Case Fatality Rate and Mortality Rate of Implicit-coded Sepsis for Both Sexes from 2017 to 2019. Table S13. Validation of Implicit-Coded Strategy for Identification of Sepsis in ICU and Non-ICU Settings abc. [file 13054_2023_4385_MOESM1_ESM.docx]

**Supplementary Appendix**

**Contents**

[Method S1. Description of Sampling Strategy and Death Data Collection in the National Mortality Surveillance System (NMSS) 2](#_Toc127558265)

[Method S2. Estimation of in-hospital sepsis case fatality rate, in-hospital sepsis mortality rate and hospitalized sepsis incidence 4](#_Toc127558266)

[Method S3. Explanations of Global and Local Moran's Index. 7](#_Toc127558267)

[Table S1. Explicit ICD-10-CM codes of sepsis 8](#_Toc127558268)

[Table S2. ICD-9-CM and ICD-10-CM Codes for Identification of Infection in NDCMS and Sepsis-related Death in NMSS 10](#_Toc127558269)

[Table S3. ICD-10-CM codes of organ dysfunction 17](#_Toc127558270)

[Table S4. ICD-9-CM codes of organ dysfunction related procedures 22](#_Toc127558271)

[Table S5. ICD-10-CM codes of Charlson Comorbidity Index 23](#_Toc127558272)

[Table S6. Demographic and Clinical Characteristics of Patients with Explicit-coded Sepsis in NDCMS from 2017 to 2019 25](#_Toc127558273)

[Table S7. Characteristics of Admissions for Implicit-Coded Sepsis From 2017 to 2019 27](#_Toc127558274)

[Table S8. Characteristics of Admissions for Explicit-Coded Sepsis From 2017 to 2019 30](#_Toc127558275)

[Table S9. Crude Province-specific Incidence, Case Fatality Rate and Mortality Rate of Implicit-coded Sepsis from 2017 to 2019 32](#_Toc127558276)

[Table S10. Standardized Province-specific Incidence, Case Fatality Rate and Mortality Rate of Implicit-coded Sepsis from 2017 to 2019 39](#_Toc127558277)

[Table S11. Association between Incidence of Implicit-coded Sepsis Hospitalization and Healthcare Resources Availability from 2017 to 2019 46](#_Toc127558278)

[Table S12. Sensitivity Analysis of Incidence, Case Fatality Rate and Mortality Rate of Implicit-coded Sepsis for Both Sexes from 2017 to 2019 47](#_Toc127558279)

[Table S13. Validation of Implicit-Coded Strategy for Identification of Sepsis in ICU and Non-ICU Settings ^abc^ 48](#_Toc127558280)

[Figure S1. Percentage of hospitals enrolled in NDCMS by province from 2017 to 2019. † ‡ 49](#_Toc127558281)

[Figure S2. Enrollment of Admissions in NDCMS 50](#_Toc127558282)

# Method S1. Description of Sampling Strategy and Death Data Collection in the National Mortality Surveillance System (NMSS)

The national mortality surveillance system was established according to the sampling strategy of the disease surveillance points system (DSPs).

First, the National Health and Family Planning Commission determined that the surveillance population should be not less than 5 million in any province that had a population greater than 10 million and was economically well developed; for other provinces, the population sample had to be at least 20% of the total population. These criteria were used to establish the number of surveillance points required in each province.

Second, all counties and districts in each province were divided into eight strata according to their degree of urbanization, population size and the crude mortality rate (total number of deaths per 1000 people per year).

Third, counties and districts in each stratum were selected as candidate surveillance points for each province in accordance with the number of surveillance points required. How representative the candidate surveillance points were of the whole province was determined using data from the 2010 census. The final surveillance points for each province were selected using an iterative process that ensured the combination of points was representative of the population of the province.

For deaths occurred in hospitals, doctors certified the cause of death and trained coders determined the underlying cause of death by applying the rules of the International Classification of Diseases. For deaths occurring outside hospital, village health workers or community hospital staff did a verbal autopsy from which doctors in these hospitals determined the underlying cause of death. Since 2008 information on individual deaths in all population catchment areas has been reported in real time using an Internet-based reporting system. In this system, information on each death is systematically validated by local – including county, prefecture, and provincial level – Centers for Disease Control and Prevention, which also check the completeness, coding and internal logic of the items reported on death certificates. Causes-of-death are subsequently reported to the national CDC, where data are consolidated.

Detailed development of the national mortality surveillance system has been described previously (Liu S, Wu X, Lopez AD, Wang L, Cai Y, Page A, et al. An integrated national mortality surveillance system for death registration and mortality surveillance, China. Bull World Health Organ 2016; 94: 46-57.)

# Method S2. Estimation of in-hospital sepsis case fatality rate, in-hospital sepsis mortality rate and hospitalized sepsis incidence

The relationship between in-hospital sepsis case fatality rate, in-hospital sepsis mortality rate and hospitalized sepsis incidence is as follows,

$$\mathrm{hospitalized}sepsis incidence=\frac{in-hospital sepsis mortality rate}{in-hospital sepsis case fatality rate}$$

and a short proof is provided below

Proof:

$$\mathrm{hospitalized}sepsis incidence=\frac{number of all hospitalized sepsis patients}{number of all individuals}=\frac{\frac{1}{number of all individuals}}{\frac{1}{number of all hospitalized sepsis patients}}=\frac{\frac{1}{number of all individuals}}{\frac{1}{number of all hospitalized sepsis patients}}\times\frac{number of sepsis-related deaths}{number of sepsis-related deaths}=\frac{\frac{number of sepsis-related deaths}{number of all individuals}}{\frac{number of sepsis-related deaths}{number of all hospitalized sepsis patients}}$$

Apprently,

$$\frac{number of sepsis-related deaths}{number of all individuals}=in-hospital sepsis mortality rate$$

$$\frac{number of sepsis-related deaths}{number of all hospitalized sepsis patients}=in-hospital sepsis case fatality rate$$

Thus,

$$\mathrm{hospitalized}sepsis incidence=\frac{in-hospital sepsis mortality rate}{in-hospital sepsis case fatality rate}$$

Detailed illustrations are also provided, and to make them more clear, we define the following notations first.

$Y$ denotes whether hospitalized sepsis patient died, if died, $Y$ of this patient is equal to 1, otherwise, equal to 0;

$Z$ denotes whether patient died in hospital and due to sepsis, similarly, $Z$ is equal to 1 if one patient meets the above two conditions, otherwise, equal to 0;

$X$ denotes whether patient is admitted to hospital and diagnosed with sepsis, $X$ is equal to 1 if the patient is hospitalized due to sepsis, otherwise equal to 0;

$A$ denotes 5 year-age group (<1, 1-4, 5-9, 10-14, 15-19, 20-24, 25-29, 30-34, 35-39, 40-44, 45-49, 50-54, 55-59, 60-64, 65-69, 70-74, 75-79, 80-84, and ≥85 years) of patient;

$S$ denotes sex (female and male) of patient;

$H$ hospital type (tertiary and secondary) patient admitted to.

In NDCMS, at each calendar year, we first categorized our included population into several strata by 5-year age, sex and hospital type. And then, at each stratum, we calculated the in-hospital sepsis case fatality rate ($P(Y|A, S, H)$) by the formula below,

$$P(Y|A, S, H)=\frac{number of sepsis-related deaths occurred in hospital at one statum of A, S, H}{number of all hospitalized sepsis patients at the stratum of A, S, H}$$

For example, the in-hospital sepsis case fatality rate of male patients with age less than 1 year and admitted to tertiary hospital can be calculated as follows.

$$P(Y|A=^{'}<1\mathrm{year}^{'}, S=^{'}male^{'}, H='\mathrm{tertiary}')=\frac{number of sepsis-related deaths occurred in hospital among male patients with age less than 1 year and admitted to tertiary hospital}{number of all hospitalized sepsis male patients with age less than 1 year and admitted to tertiary hospital}$$

Then, by the total probability formula, the 5-year age and sex specific in-hospital sepsis case fatality rates ($P\left( Y | A, S \right)$) were obtained as follows.

$$P\left( Y | A, S \right)= P\left( Y | A, S, H='\mathrm{tertiary}' \right)P\left( H='\mathrm{tertiary}'|A, S \right)+ P\left( Y | A, S, H='secondary' \right)P\left( H='secondary'|A, S \right)$$

$P\left( H='\mathrm{tertiary}'|A, S \right)$ and $P\left( H='secondary'|A, S \right)$ were extracted from China Statistical Yearbook. Thus, the in-hospital sepsis case fatality rate of male patients with age less than 1 year is like below.

$$P\left( Y | A=^{'}<1\mathrm{year}^{'}, S=^{'}male^{'} \right)= P\left( Y | A=^{'}<1\mathrm{year}^{'}, S=^{'}male^{'}, H=^{'}\mathrm{tertiary}^{'} \right)P\left( H='\mathrm{tertiary}'|A=^{'}<1\mathrm{year}^{'}, S=^{'}male^{'} \right)+P\left( Y | A=^{'}<1\mathrm{year}^{'}, S=^{'}male^{'}, H=^{'}\mathrm{secondary}^{'} \right)P\left( H='\mathrm{secondary}'|A=^{'}<1\mathrm{year}^{'}, S=^{'}male^{'} \right)$$

In NMSS, we calculated the in-hospital sepsis mortality rate ($P(Z|A,S)$) at each stratum stratified by age and sex together by the following formula,

$$P(Z|A,S)=\frac{number of sepsis-related deaths occurred in hospital at one statum of A, S}{number of all individuals at the statum of A, S}$$

For example, the in-hospital sepsis mortality rate of male patients with age less than 1 year can be calculated as follows.

$$P(Z|A=^{'}<1\mathrm{year}^{'}, S=^{'}male^{'})=\frac{number of sepsis-related deaths occurred in hospital among male patients with age less than 1 year}{number of all male individuals with age less than 1 year}$$

Once $P\left( Y | A=^{'}<1\mathrm{year}^{'}, S=^{'}male^{'} \right)$ and $P(Z|A=^{'}<1\mathrm{year}^{'}, S=^{'}male^{'})$ were obtained, the hospitalized sepsis incidence of male patients with age less than 1 year ($P\left( X | A=^{'}<1\mathrm{year}^{'}, S=^{'}male^{'} \right)$) can be calculated like below.

$$P\left( X | A=^{'}<1\mathrm{year}^{'}, S=^{'}male^{'} \right)=\frac{number of all hospitalized sepsis male patients with age less than 1 year}{number of all male individuals with age less than 1 year}=\frac{\frac{number of sepsis-related deaths occurred in hospital among male patients with age less than 1 year}{number of all male individuals with age less than 1 year}}{\frac{number of sepsis-related deaths occurred in hospital among male patients with age less than 1 year}{number of all hospitalized sepsis male patients with age less than 1 year}}=\frac{P\left( Z | A=^{'}<1\mathrm{year}^{'}, S=^{'}male^{'} \right)}{P\left( Y | A=^{'}<1\mathrm{year}^{'}, S=^{'}male^{'} \right)}$$

Similarly, in-hospital sepsis case fatality rate, in-hospital sepsis mortality rate and hospitalized sepsis incidence for patients with age in any 5-year age group and any sex can be estimated by the above process.

Finally, by using the the total probability formula again, the annual standardized annual in-hospital sepsis case fatality rate, in-hospital sepsis mortality rate and hospitalized sepsis incidence can be obtained, taking the calculation of the annual standardized hospitalized sepsis incidence for example.

$$P\left( X \right)=\sum_{A} \sum_{S} P(X|A,S)P(A, S)$$

All $P\left( A, S \right)$s were obtained from the 2010 China Census.

# Method S3. Explanations of Global and Local Moran's Index.

The Globe Moran's Index represents the spatial autocorrelation as well as the global incidence distribution pattern, and the P value of it is a measure of statistical significance and is used to resolve the null hypothesis whether the spatial distribution of incidence is owing to chance. The range value of Globe Moran's Index is between -1 and 1. A Global Moran's Index larger than 0 with P value less than 0.05 indicates spatial clustering, while a Global Moran's Index smaller than 0 with P value less than 0.05 indicates spatial dispersion.

In our anlysis, at each calendar year from 2017 to 2019, all Global Moran's Indices were larger than 0 with P value less than 0.05 (Figure 2), which means the distribution of sepsis incidence is clustered in China. Thus, Local Moran’s Index is used to further identify statistically significant spatial clusters with high or low incidence, which can be categorized into four types, high-high cluster, low-low cluster, high-low cluster and low-high cluster. The high-high and the low-low categories indicate clustering of high sepsis incidences (hot-spots) and low sepsis incidences (cold regions), respectively. Both the high-high and low-low categories indicate signicant (p≤0.05) clustering of similar incidences or positive spatial autocorrelation. The low-high cluster indicates that provinces with high sepsis incidencs surround a province with low incidence, whereas the high-low cluster indicates that provinces with low sepsis incidencs surround a province with high incidence. These are indications of spatial outliers.

# Table S1. Explicit ICD-10-CM codes of sepsis

| ICD-10-CM | Codes Description^a^ |
| --- | --- |
| A01.003 | Typhoid fever sepsis |
| A02.100 | Salmonella sepsis |
| A03.900 | Shigellosis, unspecified (shock) |
| A09.005 | Septic gastroenteritis |
| A20.7 | Septicemic plague |
| A21.7 | Generalized tularemia |
| A22.7 | Anthrax sepsis |
| A24.1 | Acute and fulminating melioidosis |
| A26.7 | Erysipelothrix sepsis |
| A27.900 | Leptospirosis |
| A28.001 | Pasteurellosis |
| A28.2 | Extraintestinal yersiniosis |
| A32.7 | Listerial sepsis |
| A38.x00x012 | Scarlet fever sepsis |
| A39.2 | Acute meningococcemia |
| A39.3 | Chronic meningococcemia |
| A39.4 | Meningococcemia， unspecified |
| A39.1 | Waterhouse |
| A40 | Streptococcal sepsis |
| A41 | Other sepsis |
| A42.7 | Actinomycotic sepsis |
| A48.3 | Toxic shock syndrome |
| A54.8 | Other gonococcal infections (sepsis) |
| A93.800x001 | Other specified arthropod-borne viral fevers [Piry virus disease] |
| A98.500 | Haemorrhagic fever with renal syndrome |
| B00.7 | Herpetic septicemia |
| B37.7 | Candidal sepsis |
| B37.6 | Candidal endocarditis |
| B49 | Unspecified mycosis (Fungemia) |
| F05.901 | Delirium, unspecified (infectious) |
| F06.800x002 | Other specified mental disorders due to brain damage and dysfunction and to physical disease (biliary infection) |
| F06.800x016 | Other specified mental disorders due to brain damage and dysfunction and to physical disease (bacillary dysentery) |
| J15.903 | Bacterial pneumonia, unspecified (severe community-acquired) |
| J18.903 | Pneumonia, unspecified (severe) |
| J95.000x001 | Sepsis of tracheostomy stoma |
| K85.800x019 | Abscess of pancreas (severe) |
| K85.817 | Abscess of pancreas (other, severe) |
| O03.300x001 | Spontaneous abortion, Incomplete, with septic shock |
| O03.800x001 | Spontaneous abortion, Complete, with septic shock |
| O04.300x004 | Medical abortion, Incomplete, with septic shock |
| O04.800x001 | Medical abortion, Complete, with septic shock |
| O04.804 | Medical abortion, later complete, with septic shock |
| O08.000 | Genital tract and pelvic infection following abortion and ectopic and molar pregnancy |
| O08.003 | Septic shock following abortion and ectopic and molar pregnancy |
| O08.200x002 | Embolism following abortion and ectopic and molar pregnancy (septic) |
| O08.200x006 | Embolism following abortion and ectopic and molar pregnancy (septicopyaemic) |
| O75.3 | Other infection during labour |
| O85 | Puerperal sepsis |
| O88.300 | Obstetric pyaemic and septic embolism |
| O98.8 | Other maternal infectious and parasitic diseases complicating pregnancy, childbirth and the puerperium |
| P36 | Bacterial sepsis of newborn |
| P37.800x002 | Other specified congenital infectious and parasitic diseases |
| R57.2 | Septic shock |
| R65.1 | Systemic inflammatory response syndrome (SIRS) due to infection with organ dysfunction |
| T80.2 | Infections following infusion, transfusion and therapeutic injection |
| T81.4 | Infection following a procedure, not elsewhere classified |
| T88.000x002 | Sepsis following immunization |

^a^ Any diagnosis with the word "severe" required complications of organ dysfunction in the official 10-digit Chinese version of International Classification of Diseases, Tenth Revision (ICD-10).

# Table S2. ICD-9-CM and ICD-10-CM Codes for Identification of Infection in NDCMS and Sepsis-related Death in NMSS

| ICD-9-CM | Codes Description | ICD-10-CM |
| --- | --- | --- |
| 001 | Cholera | A00 |
| 002 | Typhoid/paratyphoid fever | A01 |
| 003 | Other salmonella infection | A02  G01  J17  M01  M90 |
| 004 | Shigellosis | A03 |
| 005 | Other food poisoning | A05 |
| 008 | Intestinal infection not otherwise classified | A02  A04  A08 |
| 009 | Ill-defined intestinal infection | A09 |
| 010 | Primary tuberculosis | A15  A16 |
| 011 | Pulmonary tuberculosis | A15  A16 |
| 012 | Other respiratory tuberculosis | A15  A16  J38 |
| 013 | Central nervous system tuberculosis | A17 |
| 014 | Intestinal tuberculosis | A18  K93 |
| 015 | Tuberculosis of bone and joint | A18  H75 |
| 016 | Genitourinary tuberculosis | A18  N51  N74 |
| 017 | Tuberculosis not otherwise classified | A18  D77  E35  K23 |
| 018 | Military tuberculosis | A19 |
| 020 | Plague | A20 |
| 021 | Tularemia | A21 |
| 022 | Anthrax | A22 |
| 023 | Brucellosis | A23 |
| 024 | Glanders | A24 |
| 025 | Melioidosis | A24 |
| 026 | Rat-bite fever | A25 |
| 027 | Other bacterial zoonoses | A26  A28  A32 |
| 030 | Leprosy | A30 |
| 031 | Other mycobacterial disease | A31 |
| 032 | Diphtheria | A36  I41  K67  N33 |
| 033 | Whooping cough | A37 |
| 034 | Streptococcal throat/scarlet fever | A38  J02 |
| 035 | Erysipelas | A46 |
| 036 | Meningococcal infection | A39  G05  H48  M01 |
| 037 | Tetanus | A35 |
| 038 | Septicemia | A40  A41 |
| 039 | Actinomycotic infections | A42  A43  B47 |
| 040 | Other bacterial diseases | A48  K90  M60 |
| 041 | Bacterial infection in other diseases not otherwise specified | B95  B96 |
| 090 | Congenital syphilis | A50 |
| 091 | Early symptomatic syphilis | A51  R59  H22  H32  M90  K77  G01  L99 |
| 092 | Early syphilis latent | A51 |
| 093 | Cardiovascular syphilis | A52  I32  I39  I41  I79 |
| 094 | Neurosyphilis | A52  G01  G05  H32  H48  H94  I60 |
| 095 | Other late symptomatic syphilis | A52  H19  J99  K67  K77  N29  M63  M68  M90 |
| 096 | Late syphilis latent | A52 |
| 097 | Other and unspecified syphilis | A52  A53 |
| 098 | Gonococcal infections | A54  N30  N33  N39  N51  N72  N74  H19  G01  I32  I39  I52  K67 |
| 100 | Leptospirosis | A27  G01 |
| 101 | Vincent’s angina | A69 |
| 102 | Yaws | A66 |
| 103 | Pinta | A67 |
| 104 | Other spirochetal infection | A65  A69 |
| 110 | Dermatophytosis | B35 |
| 111 | Dermatomycosis not otherwise classified or specified | B36 |
| 112 | Candidiasis | A09  B37  H60 |
| 114 | Coccidioidomycosis | B38 |
| 115 | Histoplasmosis | B39  G02  H36  I32  I39  J99 |
| 116 | Blastomycotic infection | B40  B41  B48 |
| 117 | Other mycoses | B42  B43  B44  B45  B46  B47  B48 |
| 118 | Opportunistic mycoses | B48 |
| 320 | Bacterial meningitis | G00 |
| 322 | Meningitis, unspecified | G03  G05 |
| 324 | Central nervous system abscess | G06 |
| 325 | Phlebitis of intracranial sinus | G08 |
| 420 | Acute pericarditis | I30  I32 |
| 421 | Acute or subacute endocarditis | I33  I39 |
| 451 | Thrombophlebitis | I80 |
| 461 | Acute sinusitis | J01 |
| 462 | Acute pharyngitis | J02 |
| 463 | Acute tonsillitis | J03 |
| 464 | Acute laryngitis/tracheitis | J04  J05 |
| 465 | Acute upper respiratory infection of multiple sites/not otherwise specified | J06 |
| 481 | Pneumococcal pneumonia | J13 |
| 482 | Other bacterial pneumonia | J14  J15 |
| 485 | Bronchopneumonia with organism not otherwise specified | J18 |
| 486 | Pneumonia, organism not otherwise specified | J18 |
| 491.21 | Acute exacerbation of obstructive chronic bronchitis | J44.1 |
| 494 | Bronchiectasis | J47 |
| 510 | Empyema | J86 |
| 513 | Lung/mediastinum abscess | J85 |
| 540 | Acute appendicitis | K35 |
| 541 | Appendicitis not otherwise specified | K37 |
| 542 | Other appendicitis | K36 |
| 562.01 | Diverticulitis of small intestine without hemorrhage | K57.12 |
| 562.03 | Diverticulitis of small intestine with hemorrhage | K57.13 |
| 562.11 | Diverticulitis of colon without hemorrhage | K57.22 |
| 562.13 | Diverticulitis of colon with hemorrhage | K57.23 |
| 566 | Anal and rectal abscess | K61 |
| 567 | Peritonitis | K65  K67 |
| 569.5 | Intestinal abscess | K63.0 |
| 569.83 | Perforation of intestine | K63.1 |
| 572.0 | Abscess of liver | K75.0 |
| 572.1 | Portal pyema | K75.1 |
| 575.0 | Acute cholecystitis | K81.0 |
| 590 | Kidney infection | N10  N11  N12  N15 |
| 597 | Urethritis/urethral syndrome | N34 |
| 599.0 | Urinary tract infection not otherwise specified | N39.0 |
| 601 | Prostatic inflammation | N41  N51 |
| 614 | Female pelvic inflammation disease | N70  N73 |
| 615 | Uterine inflammation disease | N71 |
| 616 | Other female genital inflammation | N72  N75  N76  N77 |
| 681 | Cellulitis, finger/toe | L03 |
| 682 | Other cellulitis or abscess | L03 |
| 683 | Acute lymphadenitis | L04 |
| 686 | Other local skin infection | L08  L98 |
| 711.0 | Pyogenic arthritis | M00.9 |
| 730 | Osteomyelitis | M86  M89  M90 |
| 790.7 | Bacteremia | A49.9 |
| 996.6 | Infection or inflammation of device/graft | T82.6  T82.7  T83.5  T84.5  T84.6  T85.71  T85.78  T85.81 |
| 998.5 | Postoperative infection | T81.4 |
| 999.3 | Infectious complication of medical care not otherwise classified | T80.2 |
| Extra codes | |  |
|  | Bacterial infection of unspecified site | A49 |
|  | Typhus fever | A75 |
|  | Spotted fever [tick-borne rickettsioses] | A77 |
|  | Q fever | A78 |
|  | Other rickettsioses | A79 |
|  | Acute poliomyelitis | A80 |
|  | Atypical virus infections of central nervous system | A81 |
|  | Rabies | A82 |
|  | Mosquito-borne viral encephalitis | A83 |
|  | Tick-borne viral encephalitis | A84 |
|  | Other viral encephalitis, not elsewhere classified | A85 |
|  | Unspecified viral encephalitis | A86 |
|  | Viral meningitis | A87 |
|  | Other viral infections of central nervous system, not elsewhere classified | A88 |
|  | Unspecified viral infection of central nervous system | A89 |
|  | Dengue fever [classical dengue] | A90 |
|  | Dengue hemorrhagic fever | A91 |
|  | Other mosquito-borne viral fevers | A92 |
|  | Other arthropod-borne viral fevers, not elsewhere classified | A93 |
|  | Unspecified arthropod-borne viral fever | A94 |
|  | Yellow fever | A95 |
|  | Arenaviral hemorrhagic fever | A96 |
|  | Other viral hemorrhagic fevers, not elsewhere classified | A98 |
|  | Unspecified viral hemorrhagic fever | A99 |
|  | Other viral diseases, not elsewhere classified | B33 |
|  | Viral infection of unspecified site | B34 |
|  | Unspecified mycosis | B49 |
|  | Plasmodium falciparum malaria | B50 |
|  | Plasmodium vivax malaria | B51 |
|  | Plasmodium malariae malaria | B52 |
|  | Other specified malaria | B53 |
|  | Unspecified malaria | B54 |
|  | Leishmaniasis | B55 |
|  | African trypanosomiasis | B56 |
|  | Chagas' disease | B57 |
|  | Toxoplasmosis | B58 |
|  | Pneumocystosis | B59 |
|  | Other protozoal diseases, not elsewhere classified | B60 |
|  | Unspecified protozoal disease | B64 |
|  | Schistosomiasis [bilharziasis] | B65 |
|  | Other fluke infections | B66 |
|  | Echinococcosis | B67 |
|  | Taeniasis | B68 |
|  | Cysticercosis | B69 |
|  | Diphyllobothriasis and sparganosis | B70 |
|  | Other cestode infections | B71 |
|  | Dracunculiasis | B72 |
|  | Onchocerciasis | B73 |
|  | Filariasis | B74 |
|  | Trichinellosis | B75 |
|  | Hookworm diseases | B76 |
|  | Ascariasis | B77 |
|  | Strongyloidiasis | B78 |
|  | Trichuriasis | B79 |
|  | Enterobiasis | B80 |
|  | Other intestinal helminthiases, not elsewhere classified | B81 |
|  | Unspecified intestinal parasitism | B82 |
|  | Other helminthiases | B83 |
|  | Pediculosis and phthiriasis | B85 |
|  | Scabies | B86 |
|  | Myiasis | B87 |
|  | Other infestations | B88 |
|  | Unspecified parasitic disease | B89 |
|  | Sequelae of tuberculosis | B90 |

NDCMS = National Data Center for Medical Service; NMSS= National Mortality Surveillance System

# Table S3. ICD-10-CM codes of organ dysfunction

| System | ICD-10-CM | Codes Description |
| --- | --- | --- |
| Cardiovascular | A41.9 | Septic shock |
|  | A48.3 | Toxic shock syndrome |
|  | E86.x00 | Volume depletion |
|  | E86.x00x001 | hypovolemia |
|  | E86.x00x003 | extracellular fluid deletion |
|  | E86.x00x004 | Plasma volume depletion |
|  | E86.x00x005 | Volume depletion |
|  | E86.x01 | dehydration |
|  | I51.400x007 | Severe Myocarditis |
|  | I95.8 | Hypotension, unspecified |
|  | I95.9 | Hypotension, |
|  | I99.x00 | Other disorders of the circulatory system |
|  | I99.x01 | Circulatory disorders |
|  | R09.800x082 | Weak pulse |
|  | R57.0 | Cardiogenic shock |
|  | R57.1 | Hypovolaemic shock |
|  | R57.2 | Septic shock |
|  | R57.8 | Other shock |
|  | R57.9 | Shock, unspecified |
|  | O03.300x001 | Spontaneous abortion, Incomplete, with septic shock |
|  | O03.800x001 | Spontaneous abortion, Complete, with septic shock |
|  | O04.300x004 | Medical abortion, Incomplete, with septic shock |
|  | O04.800x001 | Medical abortion, Complete, with septic shock |
|  | O04.804 | Medical abortion, later complete, with septic shock |
|  | P29 | Cardiovascular disorders originating in the perinatal period |
|  |  |  |
| Respiratory | J80 | Acute respiratory distress syndrome |
|  | J81 | Pulmonary oedema |
|  | J95.100 | Acute pulmonary insufficiency after thoracic surgery |
|  | J95.200 | Acute pulmonary insufficiency after non-thoracic surgery |
|  | J95.800x004 | Respiratory failure after surgical procedures |
|  | J95.800x021 | Respiratory distress syndrome in adults after surgery |
|  | J96 | Acute respiratory failure with hypoxia, not elsewhere classified |
|  | J96.9 | Respiratory failure, unspecified with hypoxia |
|  | J98.4 | Other disorders of lung |
|  | J98.400x024 | Severe infection of the lungs |
|  | P22 | Respiratory distress of newborn |
|  | P28.5 | Respiratory failure of newborn |
|  | R09.0 | Asphyxia |
|  | R09.000 | suffocate |
|  | R09.2 | Respiratory arrest |
|  | R09.800x095 | Asthma |
|  | U04.9 | Severe acute respiratory syndrome [SARS], unspecified |
|  |  |  |
| Central nervous system | F05 | Delirium (not alcohol- or drug-induced) |
|  | F06.8 | Other specified mental disorders due to infection |
|  | G93.1 | Anoxic brain damage, not elsewhere classified |
|  | G93.4 | Encephalopathy, unspecified |
|  | G93.8 | Other specified disorders of brain |
|  | G93.9 | Disorder of brain, unspecified |
|  | R40 | Somnolence, stupor and coma |
|  | R41.0 | Disorientation, unspecified |
|  | R45.3 | Demoralization and apathy |
|  | R55 | Syncope and collapse |
|  |  |  |
| Renal | A98.500 | Haemorrhagic fever with renal syndrome |
|  | N17 | Acute kidney failure |
|  | N19 | Unspecified kidney failure |
|  | R34 | Anuria and oliguria |
|  | R39.2 | Extrarenal uraemia |
|  | R94.4 | Abnormal results of kidney function studies |
|  |  |  |
| Metabolic | E87.2 | Acidosis |
|  |  |  |
| Hematologic | A93.800x001 | Other specified arthropod-borne viral fevers [Piry virus disease] |
|  | D61.900x001 | Bone marrow suppression |
|  | D61.901 | Myelosuppressive anemia |
|  | D61.903 | Pancytopenia |
|  | D61.906 | Acute bone marrow hematopoietic function inhibition |
|  | D65 | Disseminated intravascular coagulation [defibrination syndrome] |
|  | D65.x00x003 | Gangrene purpura |
|  | D65.x01 | Acquired fibrinogen deficiency |
|  | D65.x02 | Acquired fibrinolytic bleeding |
|  | D65.x03 | Fibrinolytic purpura |
|  | D68.9 | Coagulation defect, unspecified |
|  | D69.000x008 | Infectious purpura |
|  | D69.000x011 | Bacterial purpura |
|  | D69.000x013 | Toxic purpura |
|  | D69.203 | purpura |
|  | D69.301 | Hemorrhagic purpura |
|  | D69.5 | Secondary thrombocytopenia |
|  | D69.501 | Secondary thrombocytopenic purpura |
|  | D69.6 | Thrombocytopenia, unspecified |
|  | D69.8 | Other specified haemorrhagic conditions |
|  | D76.200x001 | Infectious hemophagocytic syndrome |
|  | D76.200x011 | Infectious erythrophagocytic syndrome |
|  |  |  |
| Hepatic | B15.000 | Hepatitis A, accompanied by hepatic coma |
|  | B15.001 | Acute viral hepatitis A with hepatic coma |
|  | B15.002 | Acute severe severe viral hepatitis A with hepatic coma |
|  | B15.003 | Subacute severe viral hepatitis A with hepatic coma |
|  | B16.000 | Acute hepatitis B, with δ factor (co-infection) and accompanied by hepatic coma |
|  | B16.001 | Acute hepatitis B-D with hepatic coma |
|  | B16.200 | Acute hepatitis B, not accompanied by δ factor (co-infection), but with hepatic coma |
|  | B16.201 | Acute viral hepatitis B with hepatic coma |
|  | B16.202 | Subacute severe viral hepatitis B with hepatic coma |
|  | B16.203 | Acute severe hepatitis B with hepatic coma |
|  | B16.204 | Acute jaundice-free hepatitis B with hepatic coma |
|  | B16.206 | Acute severe hepatitis B with hepatic coma |
|  | B17.807 | Acute severe hepatitis hepatitis |
|  | B19.000 | Viral hepatitis, accompanied by hepatic coma |
|  | B19.000x001 | Viral hepatitis with hepatic coma |
|  | B19.001 | Acute severe viral hepatitis with hepatic coma |
|  | B19.002 | Subacute severe viral hepatitis with hepatic coma |
|  | B25.101† | Cytome hepatitis with hepatic coma |
|  | E80.600 | Bilirubin metabolism disorders, others |
|  | E80.604 | Hyperbilirubinemia |
|  | E80.700 | Bilirubin metabolism disorders |
|  | K71.100x001 | Toxic liver disease with liver failure |
|  | K71.103 | Toxic liver failure |
|  | K72.0 | Acute and subacute hepatic failure |
|  | K72.9 | Hepatic failure, unspecified |
|  | K76.7 | Hepatorenal syndrome |
|  | K76.8 | Other specified diseases of liver |
|  | K76.9 | Liver disease, unspecified |
|  | K91.825 | Liver failure after surgery |
|  |  |  |
| Others | A01.003 | Typhoid fever sepsis |
|  | A02.100 | Salmonella sepsis |
|  | A03.900 | Shigellosis, unspecified (shock) |
|  | A09.005 | Septic gastroenteritis |
|  | A20.7 | Septicemic plague |
|  | A21.7 | Generalized tularemia |
|  | A22.7 | Anthrax sepsis |
|  | A24.1 | Acute and fulminating melioidosis |
|  | A26.7 | Erysipelothrix sepsis |
|  | A27.900 | Leptospirosis |
|  | A28.001 | Pasteurellosis |
|  | A28.2 | Extraintestinal yersiniosis |
|  | A32.7 | Listerial sepsis |
|  | A38.x00x012 | Scarlet fever sepsis |
|  | A39.2 | Acute meningococcemia |
|  | A39.3 | Chronic meningococcemia |
|  | A39.4 | Meningococcemia， unspecified |
|  | A39.1 | Waterhouse |
|  | A40. | Streptococcal sepsis |
|  | A41. | Other sepsis |
|  | A42.7 | Actinomycotic sepsis |
|  | A49.103 | Streptococcal infection syndrome |
|  | A54.8 | Other gonococcal infections (sepsis) |
|  | A88.800x001 | Polio-like syndrome |
|  | B00.7 | Herpetic septicemia |
|  | B37.7 | Candidal sepsis |
|  | B37.6 | Candidal endocarditis |
|  | B49 | Unspecified mycosis (Fungemia) |
|  | D71.x00x005 | Progressive septic granulomatous disease |
|  | J15.903 | Bacterial pneumonia, unspecified (severe community-acquired) |
|  | J18.903 | Pneumonia, unspecified (severe) |
|  | J95.000x001 | Sepsis of tracheostomy stoma |
|  | O08.000x006 | Sepsis following abortion and ectopic and molar pregnancy |
|  | O08.200x002 | Embolism following abortion and ectopic and molar pregnancy (septic) |
|  | O08.200x006 | Embolism following abortion and ectopic and molar pregnancy (septicopyaemic) |
|  | O85 | Puerperal sepsis |
|  | O88.300 | Obstetric pyaemic and septic embolism |
|  | P36 | Bacterial sepsis of newborn |
|  | P37.800x002 | Other specified congenital infectious and parasitic diseases |
|  | R09.800 | Involves other specific signs and symptoms of the circulatory and respiratory systems |
|  | R65.1 | Systemic inflammatory response syndrome (SIRS) due to infection with organ dysfunction |
|  | T80.2 | Infections following infusion, transfusion and therapeutic injection |
|  | T81.4 | Infection following a procedure, not elsewhere classified |
|  | T88.000x002 | Sepsis following immunization |
|  |  |  |
| Multiple | R68.800x001 | Multiple organ failure |

# Table S4. ICD-9-CM codes of organ dysfunction related procedures

| Organ | ICD-9-CM | Codes Description |
| --- | --- | --- |
| Respiratory | 311 | Temporary tracheostomy |
| Cardiovascular | 3129 | Other permanent tracheostomy |
| Cardiovascular | 3893 | Venous catheterization, not elsewhere classified |
| Renal | 3895 | Venous catheterization for renal dialysis |
| Cardiovascular | 3897 | Central venous catheter placement with guidance |
| Cardiovascular | 3899 | Other puncture of vein |
| Renal | 3927 | Arteriovenostomy for renal dialysis |
| Renal | 3942 | Revision of arteriovenous shunt for renal dialysis |
| Others | 3965 | Extracorporeal membrane oxygenation [ECMO] |
| Renal | 3995 | Hemodialysis |
| Hepatic | 5092 | Extracorporeal hepatic assistance |
| Renal | 5498 | Peritoneal dialysis |
| Respiratory | 9390 | Non-invasive mechanical ventilation |
| Respiratory | 9391 | Intermittent positive pressure breathing [IPPB] |
| Others | 9393 | Nonmechanical methods of resuscitation |
| Respiratory | 9604 | Insertion of endotracheal tube |
| Respiratory | 9670 | Continuous invasive mechanical ventilation of unspecified duration |
| Respiratory | 9671 | Continuous invasive mechanical ventilation for less than 96 consecutive hours |
| Respiratory | 9672 | Continuous invasive mechanical ventilation for 96 consecutive hours or more |
| Others | 9960 | Cardiopulmonary resuscitation, not otherwise specified |
| Others | 9962 | Other electric countershock of heart |
| Others | 9963 | Closed chest cardiac massage |

# Table S5. ICD-10-CM codes of Charlson Comorbidity Index

| Comorbidity | ICD-10-CM |
| --- | --- |
| Myocardial infarction | I21.x, I22.x, I25.2 |
| Congestive heart failure | I09.9, I11.0, I13.0, I13.2, I25.5, I42.0, I42.5 - I42.9, I43.x, I50.x, P29.0 |
| Peripheral vascular disease | I70.x, I71.x, I73.1, I73.8, I73.9, I77.1, I79.0, I79.2, K55.1, K55.8, K55.9, Z95.8, Z95.9 |
| Cerebrovascular disease | G45.x, G46.x, H34.0, I60.x - I69.x |
| Dementia | F00.x - F03.x, F05.1, G30.x, G31.1 |
| Chronic pulmonary disease | I27.8, I27.9, J40.x - J47.x, J60.x - J67.x, J68.4, J70.1, J70.3 |
| Rheumatic disease | M05.x, M06.x, M31.5, M32.x - M34.x, M35.1, M35.3, M36.0 |
| Peptic ulcer disease | K25.x - K28.x |
| Mild liver disease | B18.x, K70.0 - K70.3, K70.9, K71.3 - K71.5, K71.7, K73.x, K74.x, K76.0, K76.2 - K76.4, K76.8, K76.9, Z94.4 |
| Diabetes without chronic complication | E10.0, E10.1, E10.6, E10.8, E10.9, E11.0, E11.1, E11.6, E11.8, E11.9, E12.0, E12.1, E12.6, E12.8, E12.9, E13.0, E13.1, E13.6, E13.8, E13.9, E14.0, E14.1, E14.6, E14.8, E14.9 |
| Diabetes with chronic complication | E10.2 - E10.5, E10.7, E11.2 - E11.5, E11.7, E12.2 - E12.5, E12.7, E13.2 - E13.5, E13.7, E14.2 - E14.5, E14.7 |
| Hemiplegia or paraplegia | G04.1, G11.4, G80.1, G80.2, G81.x, G82.x, G83.0 - G83.4, G83.9 |
| Renal disease | I12.0, I13.1, N03.2 - N03.7, N05.2 - N05.7, N18.x, N19.x, N25.0, Z49.0 - Z49.2, Z94.0, Z99.2 |
| Any malignancy, including lymphoma and leukaemia, except malignant neoplasm of skin | C00.x - C26.x, C30.x - C34.x, C37.x - C41.x, C43.x, C45.x - C58.x, C60.x - C76.x, C81.x - C85.x, C88.x, C90.x - C97.x |
| Moderate or severe liver disease | I85.0, I85.9, I86.4, I98.2, K70.4, K71.1, K72.1, K72.9, K76.5, K76.6, K76.7 |
| Metastatic solid tumour | C77.x - C80.x |
| AIDS/HIV | B20.x - B22.x, B24.x |

Note:

Charlson ME, Pompei P, Ales KL, et al. A new method of classifying prognostic comorbidity in longitudinal studies: development and validation. Journal of Chronic Diseases 1987; 40:373-383. DOI: 10.1016/0021-9681(87)90171-8

Sharabiani MT, Aylin P, Bottle A. Systematic review of comorbidity indices for administrative data. Medical Care 2012; 50(12):1109-1118. DOI: 10.1097/MLR.0b013e31825f64d0

# Table S6. Demographic and Clinical Characteristics of Patients with Explicit-coded Sepsis in NDCMS from 2017 to 2019

|  | 2017 | 2018 | 2019 |
| --- | --- | --- | --- |
| Number of patients | 595,250 | 787,432 | 1,011,166 |
| Characteristics |  |  |  |
| Age, years (SD) | 36±34 | 39±34 | 41±34 |
| Female, n (%) | 241,298 (41.0) | 321,775 (40.9) | 411,077 (40.7) |
| Charlson Comorbidity Index |  |  |  |
| none | 291,615 (49.0) | 351,750 (45.7) | 431,229 (43.5) |
| 1 | 121,585 (20.3) | 161,550 (20.5) | 201,110 (20.0) |
| 2-4 | 131,089 (23.4) | 191,995 (25.3) | 271,274 (27.1) |
| >4 | 41,961 (7.4) | 61,127 (8.5) | 101,553 (10.3) |
| Comorbidities, n (%) |  |  |  |
| Myocardial infarction | 11,567 (1.8) | 11,337 (1.9) | 21,304 (2.2) |
| Congestive heart failure | 101,031 (18.3) | 151,823 (20.2) | 221,657 (22.1) |
| Peripheral vascular disease | 11,059 (3.9) | 31,152 (3.8) | 41,432 (4.7) |
| Cerebrovascular disease | 71,595 (13.4) | 111,321 (14.0) | 161,046 (15.9) |
| Dementia | 1,306 (1.2) | 11,641 (1.4) | 11,674 (1.6) |
| Chronic pulmonary disease | 61,387 (11.3) | 101,954 (12.9) | 141,092 (14.3) |
| Rheumatic disease | 1,630 (1.1) | 1,480 (1.2) | 11,828 (1.3) |
| Peptic ulcer disease | 11,049 (2.5) | 21,606 (2.7) | 31,614 (3.0) |
| Mild liver disease | 51,729 (9.5) | 71,312 (9.9) | 101,471 (10.8) |
| Moderate or severe liver disease | 21,112 (4.4) | 41,014 (5.3) | 61,436 (6.4) |
| Diabetes without chronic complication | 61,449 (11.0) | 91,693 (11.8) | 121,677 (12.7) |
| Diabetes with chronic complication | 11,834 (2.5) | 21,872 (2.8) | 31,500 (3.0) |
| Hemiplegia or paraplegia | 1,961 (0.3) | 1,071 (0.4) | 1,322 (0.5) |
| Renal disease | 31,760 (6.7) | 51,903 (6.7) | 81,406 (8.0) |
| Malignancy | 41,165 (7.3) | 61,607 (7.7) | 81,884 (8.3) |
| Metastatic solid tumor | 1,582 (1.6) | 11,228 (1.9) | 21,670 (2.2) |
| AIDS/HIV | 1,870 (0.7) | 1,163 (0.7) | 1,923 (0.7) |

NDCMS=National Data Center for Medical Service; COPD=Chronic obstructive pulmonary disease; Malignancy=any malignancy, including lymphoma and leukemia, except malignant neoplasm of skin; HIV=human immunodeficiency virus; SD=standard deviation.

# Table S7. Characteristics of Admissions for Implicit-Coded Sepsis From 2017 to 2019

|  | 2017 | 2018 | 2019 |
| --- | --- | --- | --- |
| Numubers of hospitals, n(%) |  |  |  |
| Tertiary | 1,463 (44) | 1,491 (44) | 1,543 (44) |
| Secondary | 1,852 (56) | 1,912 (56) | 1,953 (56) |
| Numbers of adsmissions according to hospital type, n (%) |  |  |  |
| Tertiary | 2,024,918 (74.6) | 2,528,220 (73.4) | 3,310,667 (73.2) |
| Secondary | 691,081 (25.4) | 916,440 (26.6) | 1,211,299 (26.8) |
| Province, n (%) |  |  |  |
| Anhui | 58982 (2.2) | 75321 (2.2) | 85533 (1.9) |
| Beijing | 107270 (3.9) | 125075 (3.6) | 141612 (3.2) |
| Fujian | 86081 (3.2) | 98474 (2.9) | 132688 (3) |
| Gansu | 30487 (1.1) | 40644 (1.2) | 58180 (1.3) |
| Guangdong | 207666 (7.6) | 263395 (7.6) | 326824 (7.3) |
| Guangxi | 105107 (3.9) | 142469 (4.1) | 187010 (4.2) |
| Guizhou | 68927 (2.5) | 94054 (2.7) | 125319 (2.8) |
| Hainan | 26440 (1) | 33266 (1) | 43004 (1) |
| Hebei | 96720 (3.6) | 115504 (3.4) | 160607 (3.6) |
| Henan | 107074 (3.9) | 133590 (3.9) | 190966 (4.3) |
| Heilongjiang | 40711 (1.5) | 59647 (1.7) | 94687 (2.1) |
| Hubei | 117307 (4.3) | 155391 (4.5) | 213409 (4.8) |
| Hunan | 145938 (5.4) | 167781 (4.9) | 221409 (4.9) |
| Jilin | 56739 (2.1) | 70300 (2) | 87183 (1.9) |
| Jiangsu | 95713 (3.5) | 113606 (3.3) | 145616 (3.2) |
| Jiangxi | 46703 (1.7) | 70198 (2) | 107102 (2.4) |
| Liaoning | 83789 (3.1) | 106991 (3.1) | 131871 (2.9) |
| Neimenggu | 47222 (1.7) | 60660 (1.8) | 74891 (1.7) |
| Ningxia | 22043 (0.8) | 30485 (0.9) | 36833 (0.8) |
| Qinghai | 12932 (0.5) | 17255 (0.5) | 30552 (0.7) |
| Shandong | 235542 (8.7) | 286232 (8.3) | 316401 (7) |
| Shanxi | 46312 (1.7) | 62660 (1.8) | 85816 (1.9) |
| Shaanxi | 64232 (2.4) | 84638 (2.5) | 106195 (2.4) |
| Shanghai | 42739 (1.6) | 59803 (1.7) | 85595 (1.9) |
| Sichuan | 258918 (9.5) | 336428 (9.8) | 456479 (10.2) |
| Tianjin | 38026 (1.4) | 41086 (1.2) | 53943 (1.2) |
| Tibet | 1408 (0.1) | 3456 (0.1) | 4540 (0.1) |
| Xinjiang | 57562 (2.1) | 77222 (2.2) | 119601 (2.7) |
| Yunnan | 190159 (7) | 237501 (6.9) | 303429 (6.8) |
| Zhejiang | 135776 (5) | 186222 (5.4) | 237202 (5.3) |
| Chongqing | 81474 (3) | 95306 (2.8) | 127469 (2.8) |
| Organ dysfunction, n(%) |  |  |  |
| Cardiovascular | 236,800 (8.7) | 283,961 (8.2) | 349,980 (7.7) |
| Respiratory | 554,943 (20.4) | 730,084 (21.2) | 954,762 (21.1) |
| Central nervous system | 60,225 (2.2) | 67,725 (2.0) | 80,001 (1.7) |
| Renal | 221,442 (8.2) | 254,253 (7.4) | 351,439 (7.8) |
| Metabolic | 116,812 (4.3) | 123,035 (3.6) | 133,897 (3.0) |
| Hematologic | 186,572 (6.9) | 234,657 (6.8) | 323,021 (7.1) |
| Hepatic | 348,074 (12.8) | 447,340 (13.0) | 593,145 (13.1) |
| Others | 420,258 (15.5) | 516,456 (15.0) | 621,343 (13.7) |
| Multiple organs | 570,873 (21.0) | 787,149 (22.9) | 1,114,378 (24.6) |

# Table S8. Characteristics of Admissions for Explicit-Coded Sepsis From 2017 to 2019

|  | 2017 | 2018 | | 2019 | |
| --- | --- | --- | --- | --- | --- |
| Numubers of hospitals, n(%) |  |  | |  | |
| Tertiary | 1,463 (44) | 1,491 (44) | | 1,543 (44) | |
| Secondary | 1,852 (56) | 1,912 (56) | | 1,953 (56) | |
| Numbers of adsmissions according to hospital type, n (%) |  |  | |  | |
| Tertiary | 479,938 (76.3) | | 634,243 (75.2) | | 823,197 (75.4) |
| Secondary | 149,450 (23.7) | | 208,947 (24.8) | | 268,975 (24.6) |
| Province, n (%) |  | |  | |  |
| Anhui | 21067 (3.3) | | 27740 (3.3) | | 24079 (2.2) |
| Beijing | 15035 (2.4) | | 16834 (2) | | 19036 (1.7) |
| Fujian | 18079 (2.9) | | 21093 (2.5) | | 25011 (2.3) |
| Gansu | 6315 (1) | | 9140 (1.1) | | 11437 (1) |
| Guangdong | 52459 (8.3) | | 73060 (8.7) | | 87928 (8.1) |
| Guangxi | 21807 (3.5) | | 31165 (3.7) | | 41807 (3.8) |
| Guizhou | 20330 (3.2) | | 26854 (3.2) | | 36390 (3.3) |
| Hainan | 9037 (1.4) | | 11028 (1.3) | | 14255 (1.3) |
| Hebei | 10723 (1.7) | | 14699 (1.7) | | 22725 (2.1) |
| Henan | 36130 (5.7) | | 45125 (5.4) | | 59758 (5.5) |
| Heilongjiang | 4140 (0.7) | | 8047 (1) | | 14012 (1.3) |
| Hubei | 31283 (5) | | 50303 (6) | | 73808 (6.8) |
| Hunan | 48403 (7.7) | | 54134 (6.4) | | 67030 (6.1) |
| Jilin | 5882 (0.9) | | 8161 (1) | | 10606 (1) |
| Jiangsu | 23467 (3.7) | | 28781 (3.4) | | 34503 (3.2) |
| Jiangxi | 9591 (1.5) | | 14316 (1.7) | | 25542 (2.3) |
| Liaoning | 14219 (2.3) | | 19287 (2.3) | | 23673 (2.2) |
| Neimenggu | 4573 (0.7) | | 6990 (0.8) | | 9222 (0.8) |
| Ningxia | 5637 (0.9) | | 7099 (0.8) | | 7561 (0.7) |
| Qinghai | 1203 (0.2) | | 2043 (0.2) | | 3326 (0.3) |
| Shandong | 92911 (14.8) | | 113349 (13.4) | | 132161 (12.1) |
| Shanxi | 4958 (0.8) | | 7180 (0.9) | | 10780 (1) |
| Shaanxi | 15535 (2.5) | | 22695 (2.7) | | 25814 (2.4) |
| Shanghai | 7895 (1.3) | | 11081 (1.3) | | 15065 (1.4) |
| Sichuan | 55439 (8.8) | | 84032 (10) | | 120922 (11.1) |
| Tianjin | 5663 (0.9) | | 6497 (0.8) | | 8964 (0.8) |
| Tibet | 322 (0.1) | | 706 (0.1) | | 844 (0.1) |
| Xinjiang | 10931 (1.7) | | 18546 (2.2) | | 29793 (2.7) |
| Yunnan | 25646 (4.1) | | 36311 (4.3) | | 45623 (4.2) |
| Zhejiang | 36256 (5.8) | | 48573 (5.8) | | 66275 (6.1) |
| Chongqing | 14452 (2.3) | | 18321 (2.2) | | 24222 (2.2) |
| Organ dysfunction, n(%) |  | |  | |  |
| Cardiovascular | 35822 (5.7) | | 43588 (5.2) | | 48776 (4.5) |
| Central nervous system | 771 (0.1) | | 548 (0.1) | | 703 (0.1) |
| Renal | 6545 (1.0) | | 7274 (0.9) | | 5811 (0.5) |
| Hematologic | 884 (0.1) | | 1480 (0.2) | | 1656 (0.2) |
| Others | 376457 (59.8) | | 472672 (56.1) | | 575313 (52.7) |
| Multiple organs | 208909 (33.2) | | 317628 (37.7) | | 459913 (42.1) |

# Table S9. Crude Province-specific Incidence, Case Fatality Rate and Mortality Rate of Implicit-coded Sepsis from 2017 to 2019

| Year | Province | Annual sepsis incidence, per 100,000 population | In-hospital case fatality rate, %† | In-hospital mortality rate, per 100,000 population‡ |
| --- | --- | --- | --- | --- |
| 2017 | Anhui | 209.12 (197.16-221.08) | 7.63 (7.32-7.95) | 15.96 (15.33-16.59) |
| 2017 | Beijing | 262.56 (252.79-272.33) | 18.96 (18.59-19.32) | 49.77 (48.19-51.35) |
| 2017 | Fujian | 229.25 (208.97-249.53) | 2.73 (2.61-2.85) | 6.26 (5.78-6.74) |
| 2017 | Gansu | 795.21 (712.75-877.67) | 1.93 (1.76-2.09) | 15.31 (14.45-16.17) |
| 2017 | Guangdong | 333.64 (324.69-342.59) | 9.52 (9.37-9.68) | 31.77 (31.1-32.44) |
| 2017 | Guangxi | 316.34 (303.04-329.64) | 9.30 (9.12-9.48) | 29.41 (28.32-30.5) |
| 2017 | Guizhou | 397.77 (366.31-429.23) | 2.95 (2.79-3.10) | 11.72 (11.02-12.42) |
| 2017 | Hainan | 281.35 (241.48-321.22) | 3.45 (3.19-3.71) | 9.69 (8.53-10.85) |
| 2017 | Hebei | 196.09 (185.97-206.21) | 6.41 (6.24-6.57) | 12.56 (12-13.12) |
| 2017 | Henan | 265.45 (254.12-276.78) | 5.59 (5.45-5.73) | 14.84 (14.32-15.36) |
| 2017 | Heilongjiang | 380.49 (362.4-398.58) | 11.74 (11.3-12.17) | 44.65 (43.34-45.96) |
| 2017 | Hubei | 279.24 (267.06-291.42) | 7.75 (7.58-7.93) | 21.65 (20.84-22.46) |
| 2017 | Hunan | 725.33 (684.82-765.84) | 2.08 (1.99-2.16) | 15.07 (14.5-15.64) |
| 2017 | Jilin | 209.29 (197.13-221.45) | 13.15 (12.68-13.61) | 27.51 (26.25-28.77) |
| 2017 | Jiangsu | 259.62 (240.84-278.4) | 3.00 (2.83-3.16) | 7.78 (7.42-8.14) |
| 2017 | Jiangxi | 351.18 (328.93-373.43) | 5.47 (5.25-5.70) | 19.22 (18.28-20.16) |
| 2017 | Liaoning | 236.94 (225.99-247.89) | 13.25 (12.83-13.67) | 31.4 (30.34-32.46) |
| 2017 | Neimenggu | 245.9 (228.11-263.69) | 8.29 (7.92-8.65) | 20.38 (19.22-19.22) |
| 2017 | Ningxia | 212.52 (185.4-239.64) | 4.04 (3.79-4.30) | 8.59 (7.64-9.54) |
| 2017 | Qinghai | 476.55 (409.13-543.97) | 3.91 (3.56-4.26) | 18.64 (16.61-20.67) |
| 2017 | Shandong | 268.87 (259.42-278.32) | 5.98 (5.88-6.09) | 16.09 (15.6-16.58) |
| 2017 | Shanxi | 153.63 (139.41-167.85) | 5.26 (5.01-5.50) | 8.08 (7.43-8.73) |
| 2017 | Shaanxi | 339.07 (314.75-363.39) | 5.37 (5.19-5.54) | 18.2 (17.04-19.36) |
| 2017 | Shanghai | 207.85 (200.44-215.26) | 22.46 (22.04-22.88) | 46.69 (45.27-48.11) |
| 2017 | Sichuan | 547.83 (529.34-566.32) | 6.97 (6.81-7.14) | 38.2 (37.28-39.12) |
| 2017 | Tianjin | 416.53 (384.19-448.87) | 7.62 (7.12-8.11) | 31.73 (30.39-33.07) |
| 2017 | Tibet | 323.57 (237.57-409.57) | 3.96 (3.23-4.70) | 12.82 (10.38-15.26) |
| 2017 | Xinjiang | 399.98 (376.53-423.43) | 7.56 (7.33-7.79) | 30.24 (28.71-31.77) |
| 2017 | Yunnan | 346.89 (327.12-366.66) | 4.25 (4.15-4.35) | 14.75 (13.98-15.53) |
| 2017 | Zhejiang | 233.59 (220.31-246.87) | 5.17 (5.02-5.31) | 12.07 (11.47-12.67) |
| 2017 | Chongqing | 466.75 (446.28-487.22) | 6.85 (6.66-7.03) | 31.96 (30.85-33.07) |
| 2018 | Anhui | 198.26 (187.4-209.12) | 7.90 (7.60-8.20) | 15.67 (15.05-16.29) |
| 2018 | Beijing | 309.31 (298.29-320.33) | 17.79 (17.45-18.13) | 55.03 (53.38-56.68) |
| 2018 | Fujian | 243.31 (222.76-263.86) | 2.75 (2.64-2.87) | 6.7 (6.21-7.19) |
| 2018 | Gansu | 846 (765.28-926.72) | 1.81 (1.67-1.95) | 15.32 (14.46-16.18) |
| 2018 | Guangdong | 382.07 (372.6-391.54) | 9.22 (9.09-9.36) | 35.24 (34.54-35.94) |
| 2018 | Guangxi | 256.32 (244.84-267.8) | 9.26 (9.10-9.42) | 23.73 (22.75-24.71) |
| 2018 | Guizhou | 368.15 (341.88-394.42) | 3.14 (3.02-3.26) | 11.55 (10.86-12.24) |
| 2018 | Hainan | 231.15 (197.06-265.24) | 3.45 (3.22-3.68) | 7.98 (6.93-9.03) |
| 2018 | Hebei | 211.56 (201.1-222.02) | 6.31 (6.16-6.46) | 13.35 (12.77-13.93) |
| 2018 | Henan | 237.17 (227.55-246.79) | 6.20 (6.07-6.33) | 14.7 (14.19-15.21) |
| 2018 | Heilongjiang | 413.58 (396.15-431.01) | 10.99 (10.66-11.33) | 45.47 (44.15-46.79) |
| 2018 | Hubei | 268.96 (257.63-280.29) | 8.08 (7.93-8.23) | 21.73 (20.91-22.55) |
| 2018 | Hunan | 651.61 (616.76-686.46) | 2.14 (2.06-2.22) | 13.94 (13.4-14.48) |
| 2018 | Jilin | 231.09 (218.56-243.62) | 12.11 (11.75-12.46) | 27.98 (26.7-29.26) |
| 2018 | Jiangsu | 273.27 (254.82-291.72) | 2.83 (2.69-2.96) | 7.72 (7.36-8.08) |
| 2018 | Jiangxi | 342.98 (323.35-362.61) | 5.97 (5.78-6.17) | 20.49 (19.53-21.45) |
| 2018 | Liaoning | 272.69 (261.29-284.09) | 11.97 (11.66-12.28) | 32.64 (31.56-33.72) |
| 2018 | Neimenggu | 264.54 (246.54-282.54) | 8.13 (7.82-8.43) | 21.5 (20.28-22.72) |
| 2018 | Ningxia | 250.92 (220.52-281.32) | 3.68 (3.47-3.90) | 9.24 (8.26-10.22) |
| 2018 | Qinghai | 421.22 (360.67.481.77)-) | 3.90 (3.57-4.23) | 16.43 (14.53-18.33) |
| 2018 | Shandong | 328.9 (318.74-339.06) | 6.38 (6.28-6.47) | 20.97 (20.4-21.54) |
| 2018 | Shanxi | 178.91 (163.65-194.17) | 5.09 (4.88-5.29) | 9.1 (8.42-9.78) |
| 2018 | Shaanxi | 356.46 (331.6-381.32) | 5.15 (5.00-5.31) | 18.37 (17.21-19.53) |
| 2018 | Shanghai | 274.64 (265.49-283.79) | 19.61 (19.27-19.95) | 53.86 (52.33-55.39) |
| 2018 | Sichuan | 572.23 (554.34-590.12) | 6.87 (6.73-7.00) | 39.29 (38.36-40.22) |
| 2018 | Tianjin | 376.32 (348.89-403.75) | 8.88 (8.35-9.41) | 33.43 (32.04-34.82) |
| 2018 | Tibet | 467.33 (358.8-575.86) | 2.87 (2.46-3.28) | 13.42 (10.97-15.87) |
| 2018 | Xinjiang | 371.03 (350.93-391.13) | 8.76 (8.55-8.98) | 32.51 (30.94-34.08) |
| 2018 | Yunnan | 313.8 (295.52-332.08) | 4.29 (4.20-4.38) | 13.46 (12.73-14.19) |
| 2018 | Zhejiang | 276.27 (261.99-290.55) | 5.05 (4.93-5.17) | 13.94 (13.3-14.58) |
| 2018 | Chongqing | 446.76 (427.33-466.19) | 6.83 (6.66-7.00) | 30.52 (29.43-31.61) |
| 2019 | Anhui | 183.08 (173.94-192.22) | 8.52 (8.26-8.78) | 15.6 (14.98-16.22) |
| 2019 | Beijing | 386.45 (373.7-399.2) | 17.03 (16.72-17.34) | 65.81 (64.01-67.61) |
| 2019 | Fujian | 280.88 (259.05-302.71) | 2.71 (2.61-2.81) | 7.61 (7.09-8.13) |
| 2019 | Gansu | 826.92 (761.07-892.77) | 2.05 (1.93-2.17) | 16.98 (16.08-17.88) |
| 2019 | Guangdong | 414.11 (404.33-423.89) | 8.94 (8.82-9.06) | 37.03 (36.31-37.75) |
| 2019 | Guangxi | 271.14 (259.16-283.12) | 8.72 (8.58-8.86) | 23.64 (22.66-24.62) |
| 2019 | Guizhou | 488.52 (457.97-519.07) | 3.01 (2.91-3.12) | 14.73 (13.95-15.51) |
| 2019 | Hainan | 273.75 (236.19-311.31) | 3.28 (3.08-3.47) | 8.97 (7.86-10.08) |
| 2019 | Hebei | 239.22 (228.17-250.27) | 6.20 (6.07-6.33) | 14.82 (14.21-15.43) |
| 2019 | Henan | 239.05 (229.89-248.21) | 6.50 (6.38-6.61) | 15.53 (15-16.06) |
| 2019 | Heilongjiang | 430.49 (414.19-446.79) | 10.96 (10.69-11.23) | 47.19 (45.84-48.54) |
| 2019 | Hubei | 347.85 (334.1-361.6) | 7.04 (6.92-7.16) | 24.48 (23.61-25.35) |
| 2019 | Hunan | 769.25 (730.78-807.72) | 1.97 (1.90-2.03) | 15.13 (14.56-15.7) |
| 2019 | Jilin | 274.95 (261.2-288.7) | 11.38 (11.09-11.66) | 31.28 (29.93-32.63) |
| 2019 | Jiangsu | 263.7 (247.69-279.71) | 3.02 (2.90-3.14) | 7.96 (7.6-8.32) |
| 2019 | Jiangxi | 453.8 (430.62-476.98) | 5.43 (5.28-5.58) | 24.66 (23.6-25.72) |
| 2019 | Liaoning | 248.22 (238.46-257.98) | 12.76 (12.5-13.02) | 31.68 (30.61-32.75) |
| 2019 | Neimenggu | 281.29 (263.29-299.29) | 7.91 (7.65-8.17) | 22.25 (21.02-23.48) |
| 2019 | Ningxia | 238.06 (209.11-267.01) | 3.66 (3.46-3.86) | 8.71 (7.76-9.66) |
| 2019 | Qinghai | 432.36 (373.81-490.91) | 3.72 (3.46-3.98) | 16.09 (14.22-17.96) |
| 2019 | Shandong | 311.92 (302.52-321.32) | 6.62 (6.54-6.71) | 20.66 (20.1-21.22) |
| 2019 | Shanxi | 218.88 (201.55-236.21) | 4.65 (4.48-4.81) | 10.17 (9.45-10.89) |
| 2019 | Shaanxi | 316.17 (294.02-338.32) | 5.47 (5.33-5.61) | 17.3 (16.17-18.43) |
| 2019 | Shanghai | 336.03 (325.34-346.72) | 17.04 (16.77-17.32) | 57.27 (55.7-58.84) |
| 2019 | Sichuan | 680.84 (661.27-700.41) | 6.31 (6.20-6.43) | 42.99 (42.02-43.96) |
| 2019 | Tianjin | 403.43 (377.73-428.28) | 9.53 (9.04-10.01) | 38.44 (36.95-39.93) |
| 2019 | Tibet | 182.42 (95.57-269.27) | 6.55 (3.69-9.41) | 11.96 (9.68-14.24) |
| 2019 | Xinjiang | 420.26 (398.78-441.74) | 8.19 (8.02-8.35) | 34.41 (32.8-36.02) |
| 2019 | Yunnan | 336.37 (316.96-355.78) | 4.01 (3.93-4.09) | 13.48 (12.75-14.21) |
| 2019 | Zhejiang | 317.98 (302.33-333.63) | 4.73 (4.63-4.84) | 15.05 (14.38-15.72) |
| 2019 | Chongqing | 557.02 (534.96-579.08) | 6.48 (6.33-6.62) | 36.08 (34.9-37.26) |

Data in parentheses are 95% confidence intervals. HAQ= Healthcare Access and Quality.

Data of HAQ index were adapted from: Fullman N, Yearwood J, Abay SM, et al. Measuring performance on the Healthcare Access and Quality Index for 195 countries and territories and selected subnational locations: a systematic analysis from the Global Burden of Disease Study 2016. The Lancet 2018;391(10136):2236-2271.

† Estimated from National Data Center for Medical Service;

‡ Estimated from National Mortality Surveillance System.

# Table S10. Standardized Province-specific Incidence, Case Fatality Rate and Mortality Rate of Implicit-coded Sepsis from 2017 to 2019

| Year | Province | Annual sepsis incidence, per 100,000 population | In-hospital case fatality rate, %† | In-hospital mortality rate, per 100,000 population‡ |
| --- | --- | --- | --- | --- |
| 2017 | Anhui | 208.86 (187.61-230.11) | 5.81 (5.27-6.35) | 12.13 (11.64-12.62) |
| 2017 | Beijing | 501.49 (462.03-540.95) | 8.29 (7.69-8.88) | 41.56 (40.23-42.89) |
| 2017 | Fujian | 192.11 (164.49-219.73) | 3.02 (2.65-3.38) | 5.80 (5.35-6.25) |
| 2017 | Gansu | 928.59 (774.34-1082.84) | 2.01 (1.70-2.32) | 18.65 (17.56-19.74) |
| 2017 | Guangdong | 332.91 (318.4-347.42) | 8.6 (8.27-8.92) | 28.61 (27.99-29.23) |
| 2017 | Guangxi | 301.73 (283.72-319.74) | 8.9 (8.49-9.31) | 26.85 (25.84-27.86) |
| 2017 | Guizhou | 414.87 (368.43-461.31) | 2.75 (2.49-3.00) | 11.39 (10.7-12.08) |
| 2017 | Hainan | 261.24 (198.71-323.77) | 3.46 (2.75-4.17) | 9.04 (7.94-10.14) |
| 2017 | Hebei | 265.06 (242.67-287.45) | 4.32 (4.01-4.63) | 11.45 (10.94-11.96) |
| 2017 | Henan | 289.59 (269.75-309.43) | 4.61 (4.34-4.89) | 13.36 (12.89-13.83) |
| 2017 | Heilongjiang | 596.15 (531.17-661.13) | 6.14 (5.49-6.78) | 36.59 (35.5-37.68) |
| 2017 | Hubei | 338.67 (315.96-361.38) | 5.12 (4.84-5.40) | 17.34 (16.69-17.99) |
| 2017 | Hunan | 560.54 (500.47-620.61) | 2.12 (1.91-2.33) | 11.87 (11.42-12.32) |
| 2017 | Jilin | 270.69 (236.14-305.24) | 8.22 (7.25-9.20) | 22.26 (21.22-23.3) |
| 2017 | Jiangsu | 150.4 (123.75-177.05) | 3.76 (3.12-4.40) | 5.65 (5.38-5.92) |
| 2017 | Jiangxi | 286.72 (256-317.44) | 5.92 (5.36-6.49) | 16.98 (16.15-17.81) |
| 2017 | Liaoning | 304.62 (276.72-332.52) | 6.93 (6.34-7.52) | 21.1 (20.38-21.82) |
| 2017 | Neimenggu | 318.21 (264.46-371.96) | 6.72 (5.66-7.79) | 21.4 (20.15-22.65) |
| 2017 | Ningxia | 311.94 (254.78-369.1) | 3.63 (3.11-4.15) | 11.33 (10.03-12.63) |
| 2017 | Qinghai | 705.59 (569.47-841.71) | 3.25 (2.74-3.76) | 22.94 (20.35-25.53) |
| 2017 | Shandong | 176.89 (166.58-187.2) | 6.51 (6.19-6.83) | 11.52 (11.16-11.88) |
| 2017 | Shanxi | 180.63 (151.39-209.87) | 4.05 (3.48-4.62) | 7.32 (6.73-7.91) |
| 2017 | Shaanxi | 462.88 (414.77-510.99) | 3.90 (3.58-4.21) | 18.03 (16.87-19.19) |
| 2017 | Shanghai | 300.42 (270.6-330.24) | 9.22 (8.35-10.08) | 27.69 (26.8-28.58) |
| 2017 | Sichuan | 614.6 (575.9-653.3) | 4.47 (4.21-4.73) | 27.47 (26.8-28.14) |
| 2017 | Tianjin | 642.64 (573.17-712.11) | 3.55 (3.20-3.91) | 22.84 (21.86-23.82) |
| 2017 | Tibet | 342.96 (245.09-440.83) | 6.47 (5.16-7.77) | 22.17 (17.7-26.64) |
| 2017 | Xinjiang | 663.18 (608.13-718.23) | 5.95 (5.57-6.33) | 39.47 (37.4-41.54) |
| 2017 | Yunnan | 491.03 (457.19-524.87) | 3.13 (2.99-3.27) | 15.36 (14.56-16.16) |
| 2017 | Zhejiang | 208.86 (188.08-229.64) | 3.98 (3.64-4.32) | 8.31 (7.89-8.73) |
| 2017 | Chongqing | 516.54 (474.15-558.93) | 4.81 (4.46-5.17) | 24.87 (23.98-25.76) |
| 2018 | Anhui | 163.32 (143.39-183.25) | 6.59 (5.83-7.34) | 10.76 (10.32-11.2) |
| 2018 | Beijing | 487.73 (437.22-538.24) | 9.00 (8.11-9.90) | 43.92 (42.59-45.25) |
| 2018 | Fujian | 203.11 (176.88-229.34) | 2.99 (2.68-3.31) | 6.08 (5.63-6.53) |
| 2018 | Gansu | 778.97 (643.08-914.86) | 2.09 (1.75-2.43) | 16.28 (15.34-17.22) |
| 2018 | Guangdong | 388.43 (373.29-403.57) | 8.01 (7.75-8.28) | 31.12 (30.49-31.75) |
| 2018 | Guangxi | 249.04 (234.73-263.35) | 8.62 (8.28-8.96) | 21.48 (20.58-22.38) |
| 2018 | Guizhou | 409.02 (371.36-446.68) | 2.74 (2.55-2.93) | 11.22 (10.53-11.91) |
| 2018 | Hainan | 206.17 (164.18-248.16) | 3.96 (3.36-4.57) | 8.17 (7.07-9.27) |
| 2018 | Hebei | 262.48 (238.33-286.63) | 4.33 (3.98-4.68) | 11.37 (10.87-11.87) |
| 2018 | Henan | 275.29 (257.52-293.06) | 4.68 (4.42-4.93) | 12.88 (12.43-13.33) |
| 2018 | Heilongjiang | 544.12 (467.48-620.76) | 6.34 (5.47-7.21) | 34.49 (33.47-35.51) |
| 2018 | Hubei | 306.49 (286.93-326.05) | 5.40 (5.13-5.68) | 16.56 (15.93-17.19) |
| 2018 | Hunan | 528.22 (464.93-591.51) | 2.00 (1.77-2.22) | 10.56 (10.14-10.98) |
| 2018 | Jilin | 331.86 (303.44-360.28) | 6.46 (6.00-6.92) | 21.44 (20.44-22.44) |
| 2018 | Jiangsu | 206.80 (179.42-234.18) | 2.67 (2.34-3.00) | 5.52 (5.25-5.79) |
| 2018 | Jiangxi | 304.98 (275.21-334.75) | 5.77 (5.28-6.26) | 17.60 (16.77-18.43) |
| 2018 | Liaoning | 315.53 (285.4-345.66) | 6.72 (6.12-7.32) | 21.20 (20.48-21.92) |
| 2018 | Neimenggu | 412.74 (367.57-457.91) | 5.00 (4.54-5.47) | 20.65 (19.46-21.84) |
| 2018 | Ningxia | 365.44 (303.64-427.24) | 3.07 (2.67-3.46) | 11.21 (9.99-12.43) |
| 2018 | Qinghai | 645.21 (524.84-765.58) | 3.02 (2.59-3.45) | 19.49 (17.16-21.82) |
| 2018 | Shandong | 219.44 (208.46-230.42) | 6.21 (5.95-6.46) | 13.62 (13.24-14.00) |
| 2018 | Shanxi | 196.12 (167.34-224.90) | 3.94 (3.45-4.44) | 7.73 (7.15-8.31) |
| 2018 | Shaanxi | 478.85 (431.79-525.91) | 3.55 (3.28-3.82) | 17.00 (15.92-18.08) |
| 2018 | Shanghai | 411.64 (379.86-443.42) | 7.25 (6.73-7.76) | 29.83 (28.93-30.73) |
| 2018 | Sichuan | 662.1 (627.80-696.40) | 4.01 (3.82-4.19) | 26.54 (25.89-27.19) |
| 2018 | Tianjin | 355.25 (304.08-406.42) | 6.91 (5.96-7.86) | 24.56 (23.52-25.60) |
| 2018 | Tibet | 371.89 (275.18-468.60) | 5.76 (4.77-6.75) | 21.42 (17.24-25.60) |
| 2018 | Xinjiang | 584.45 (542.82-626.08) | 7.13 (6.77-7.49) | 41.66 (39.57-43.75) |
| 2018 | Yunnan | 442.39 (412.52-472.26) | 3.02 (2.90-3.14) | 13.36 (12.63-14.09) |
| 2018 | Zhejiang | 220.09 (200.26-239.92) | 3.91 (3.62-4.21) | 8.62 (8.21-9.03) |
| 2018 | Chongqing | 485.55 (448.90-522.20) | 4.62 (4.32-4.93) | 22.45 (21.62-23.28) |
| 2019 | Anhui | 188.74 (170.12-207.36) | 6.13 (5.58-6.68) | 11.57 (11.10-12.04) |
| 2019 | Beijing | 659.54 (592.37-726.71) | 8.10 (7.30-8.89) | 53.39 (51.92-54.86) |
| 2019 | Fujian | 268.81 (236.94-300.68) | 2.62 (2.37-2.87) | 7.05 (6.56-7.54) |
| 2019 | Gansu | 727.07 (625.39-828.75) | 2.18 (1.90-2.46) | 15.83 (14.97-16.69) |
| 2019 | Guangdong | 485.57 (467.67-503.47) | 6.95 (6.73-7.16) | 33.73 (33.07-34.39) |
| 2019 | Guangxi | 265.90 (251.33-280.47) | 8.05 (7.77-8.33) | 21.41 (20.51-22.31) |
| 2019 | Guizhou | 560.04 (510.90-609.18) | 2.64 (2.46-2.82) | 14.78 (13.98-15.58) |
| 2019 | Hainan | 298.99 (240.5-357.48) | 2.92 (2.48-3.36) | 8.74 (7.64-9.84) |
| 2019 | Hebei | 297.19 (270.92-323.46) | 4.19 (3.86-4.52) | 12.45 (11.93-12.97) |
| 2019 | Henan | 314.71 (296.88-332.54) | 4.50 (4.29-4.70) | 14.15 (13.66-14.64) |
| 2019 | Heilongjiang | 640.06 (588.45-691.67) | 5.56 (5.14-5.98) | 35.60 (34.56-36.64) |
| 2019 | Hubei | 400.23 (375.81-424.65) | 4.85 (4.62-5.09) | 19.43 (18.73-20.13) |
| 2019 | Hunan | 620.20 (556.26-684.14) | 1.83 (1.66-2.01) | 11.36 (10.93-11.79) |
| 2019 | Jilin | 434.26 (401.4-467.12) | 5.70 (5.35-6.05) | 24.77 (23.67-25.87) |
| 2019 | Jiangsu | 224.07 (196.38-251.76) | 2.59 (2.29-2.88) | 5.8 (5.53-6.07) |
| 2019 | Jiangxi | 408.31 (374.75-441.87) | 5.25 (4.88-5.61) | 21.42 (20.49-22.35) |
| 2019 | Liaoning | 316.03 (294.14-337.92) | 6.47 (6.09-6.86) | 20.46 (19.75-21.17) |
| 2019 | Neimenggu | 401.21 (342.67-459.75) | 5.21 (4.51-5.91) | 20.91 (19.74-22.08) |
| 2019 | Ningxia | 319.08 (265.37-372.79) | 3.08 (2.70-3.47) | 9.84 (8.74-10.94) |
| 2019 | Qinghai | 630.19 (516.64-743.74) | 2.87 (2.48-3.25) | 18.07 (15.9-20.24) |
| 2019 | Shandong | 228.45 (217.99-238.91) | 5.81 (5.60-6.02) | 13.28 (12.91-13.65) |
| 2019 | Shanxi | 263.72 (228.38-299.06) | 3.35 (2.97-3.73) | 8.84 (8.21-9.47) |
| 2019 | Shaanxi | 445.94 (403.97-487.91) | 3.39 (3.16-3.62) | 15.12 (14.12-16.12) |
| 2019 | Shanghai | 568.76 (525.31-612.21) | 6.33 (5.88-6.77) | 35.98 (34.95-37.01) |
| 2019 | Sichuan | 827.14 (788.76-865.52) | 3.56 (3.42-3.71) | 29.46 (28.78-30.14) |
| 2019 | Tianjin | 566.49 (511.83-621.15) | 5.28 (4.81-5.74) | 29.89 (28.71-31.07) |
| 2019 | Tibet | 244.67 (182.61-306.73) | 6.92 (5.90-7.95) | 16.94 (13.45-20.43) |
| 2019 | Xinjiang | 737.07 (689.34-784.80) | 6.03 (5.77-6.29) | 44.46 (42.31-46.61) |
| 2019 | Yunnan | 502.91 (469.52-536.30) | 2.65 (2.55-2.75) | 13.33 (12.60-14.06) |
| 2019 | Zhejiang | 299.73 (275.68-323.78) | 3.35 (3.13-3.58) | 10.06 (9.60-10.52) |
| 2019 | Chongqing | 649.68 (605.27-694.09) | 3.83 (3.60-4.05) | 24.85 (24.02-25.68) |

Data in parentheses are 95% confidence intervals. HAQ= Healthcare Access and Quality.

† Estimated from National Data Center for Medical Service;

‡ Estimated from National Mortality Surveillance System.

# Table S11. Association between Incidence of Implicit-coded Sepsis Hospitalization and Healthcare Resources Availability from 2017 to 2019

|  | 2017 |  | 2018 |  | 2019 |  |
| --- | --- | --- | --- | --- | --- | --- |
|  | Regression Coefficient‡ | P value | Regression Coefficient‡ | P value | Regression Coefficient‡ | P value |
| Doctors per 10,000 population † | -28.69 (-50.59--6.79) | 0.010 | -10.81 (-26.29-4.67) | 0.171 | -21.57 (-39.94--3.2) | 0.021 |
| Registered nurses per 10,000 population † | -10.29 (-25.85-5.27) | 0.195 | -10.16 (-21.4-1.08) | 0.077 | -2.34 (-16.43-11.75) | 0.745 |
| Hospital beds per 10,000 population † | 10.94 (2.46-19.42) | 0.012 | 10.05 (4.74-15.36) | <0.001 | 12.72 (6.12-19.32) | <0.001 |
| Disposable income per capita (per 1000 yuan RMB) † | 18.84 (5.97-31.71) | 0.004 | 9.69 (1.64-17.74) | 0.018 | 14.74 (5.25-24.23) | 0.002 |

† Data obtained from National Bureau of Statistics of China (<http://www.stats.gov.cn>).

‡ Regression model was fitted by excluding data from Beijing, Shanghai due to the high proportion of non-local resident hospitalizations.

# Table S12. Sensitivity Analysis of Incidence, Case Fatality Rate and Mortality Rate of Implicit-coded Sepsis for Both Sexes from 2017 to 2019

|  | 2017 | 2018 | 2019 |
| --- | --- | --- | --- |
| **Crude** |  |  |  |
| Annual sepsis incidence, per 100,000 population | 308.56 (305.81-311.31) | 332.76 (329.97-335.55) | 371.36 (368.43-374.29) |
| In-hospital case fatality rate, % | 6.83 (6.80-6.87) | 6.78 (6.75-6.81) | 6.52 (6.49-6.54) |
| In-hospital mortality rate, per 100,000 population | 21.08 (20.92-21.24) | 22.56 (22.40-22.72) | 24.21 (24.04-24.38) |
| **Standardized** |  |  |  |
| Annual sepsis incidence, per 100,000 population | 341.79 (328.24-355.34) | 359.37 (345.50-373.24) | 430.30 (414.67-445.93) |
| In-hospital case fatality rate, % | 5.07 (5.00-5.13) | 4.88 (4.83-4.94) | 4.45 (4.41-4.50) |
| In-hospital mortality rate, per 100,000 population | 17.32 (16.67-17.97) | 17.55 (16.90-18.20) | 19.16 (18.49-19.83) |

Data in parentheses are 95% confidence intervals. In-hospital sepsis case fatality rate= the proportion of patients with sepsis who die in hospitals among all hospitalized sepsis patients over one calendar year; In-hospital sepsis mortality rate = the proportion of all in-hospital sepsis-related deaths among the whole population over one calendar year.

# Table S13. Validation of Implicit-Coded Strategy for Identification of Sepsis in ICU and Non-ICU Settings ^abc^

| **Patients** | **SP (95% CI)** | **SE (95% CI)** | **PPV (95% CI)** | **NPV (95% CI)** | **AUC (95% CI)** |
| --- | --- | --- | --- | --- | --- |
| **No-ICU (n=409)** | 80.00(73.67-85.68) | 64.19(57.57-70.33) | 80.33(73.67-85.68) | 63.72(57.04-69.92) | 72.10(67.09-77.11) |
| **ICU (n=138)** | 88.57(72.32-96.27) | 84.47(75.70-90.59) | 95.60(88.50-98.58) | 65.96(50.60-78.72) | 86.52(79.17-93.86) |
| **ICU and non-ICU (n=547)** | 81.39(75.41-86.23) | 70.48(65.20-75.27) | 85.40(80.53-89.25) | 64.10(58.07-69.74) | 75.94(71.77-80.11) |

^a^The validation was performed based on a retrospective analysis of a prospective, single-center, cohort study which was designed to assess the diagnostic value of qSOFA for sepsis in general ward and Intensive Care Medicine.

^b^ All sepsis was defined according to the Third International Consensus Definitions for Sepsis (Sepsis-3), i.e. a change in SOFA score ≥2 points consequent to an infection.

^c^ Values expressed as % (95% CI) unless otherwise indicated.

Abbreviations: SP, Specificity; SE, Sensitivity; PPV, positive predictive value; NPV, negative predictive value; CI, confidence interval; AUC, area under curve


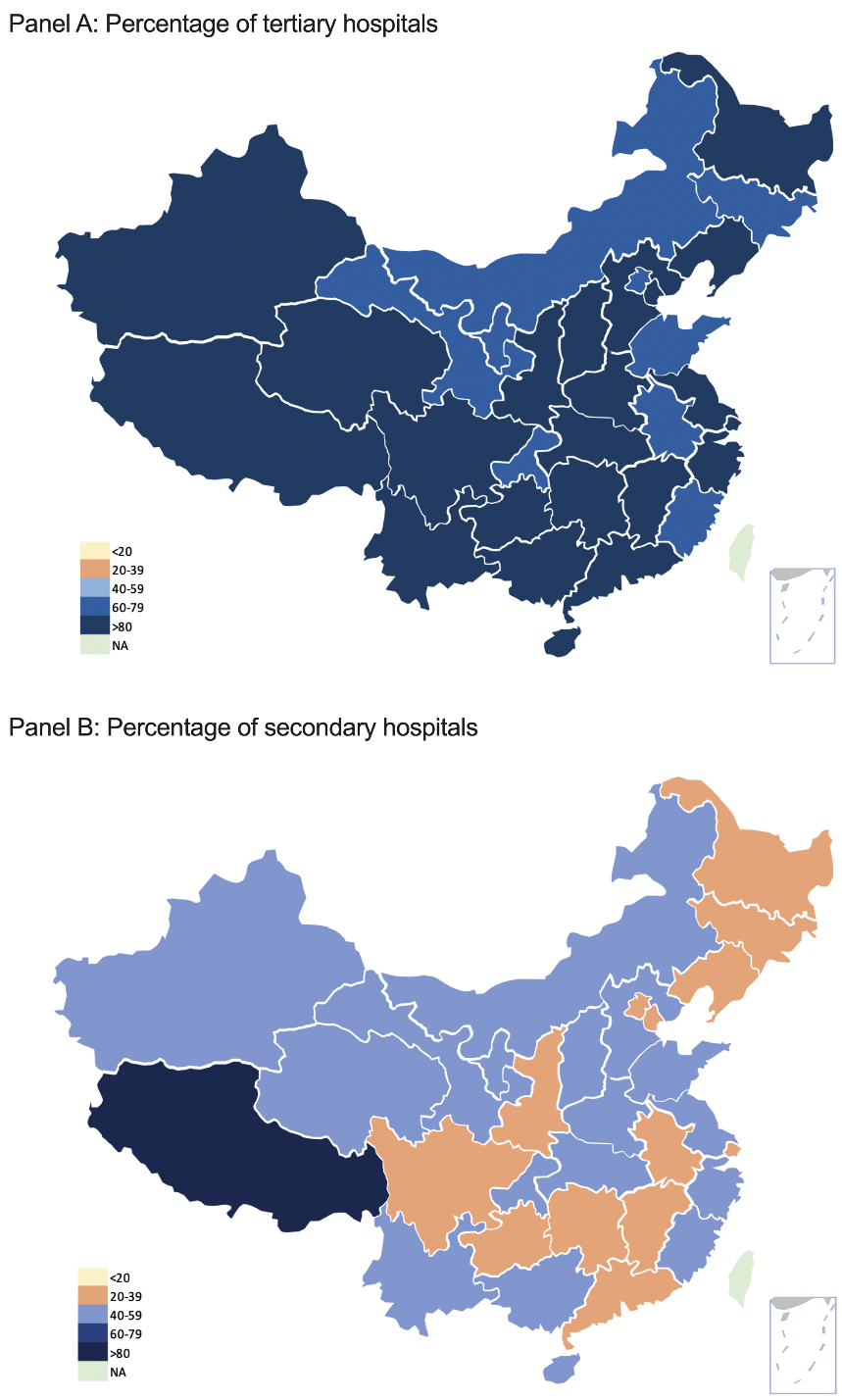


# Figure S1. Percentage of hospitals enrolled in NDCMS by province from 2017 to 2019. † ‡

Panel A: the percentage of tertiary hospitals enrolled in NDCMS by province; Panel B: the percentage of secondary hospitals enrolled in NDCMS by province

NDCMS = National Data Center for Medical Service, NA Not Available.

† The number of all hospitals and admissions in China was obtained from the China Health Statistics Yearbook 2020.

‡ All numbers were reported as percentage.

#
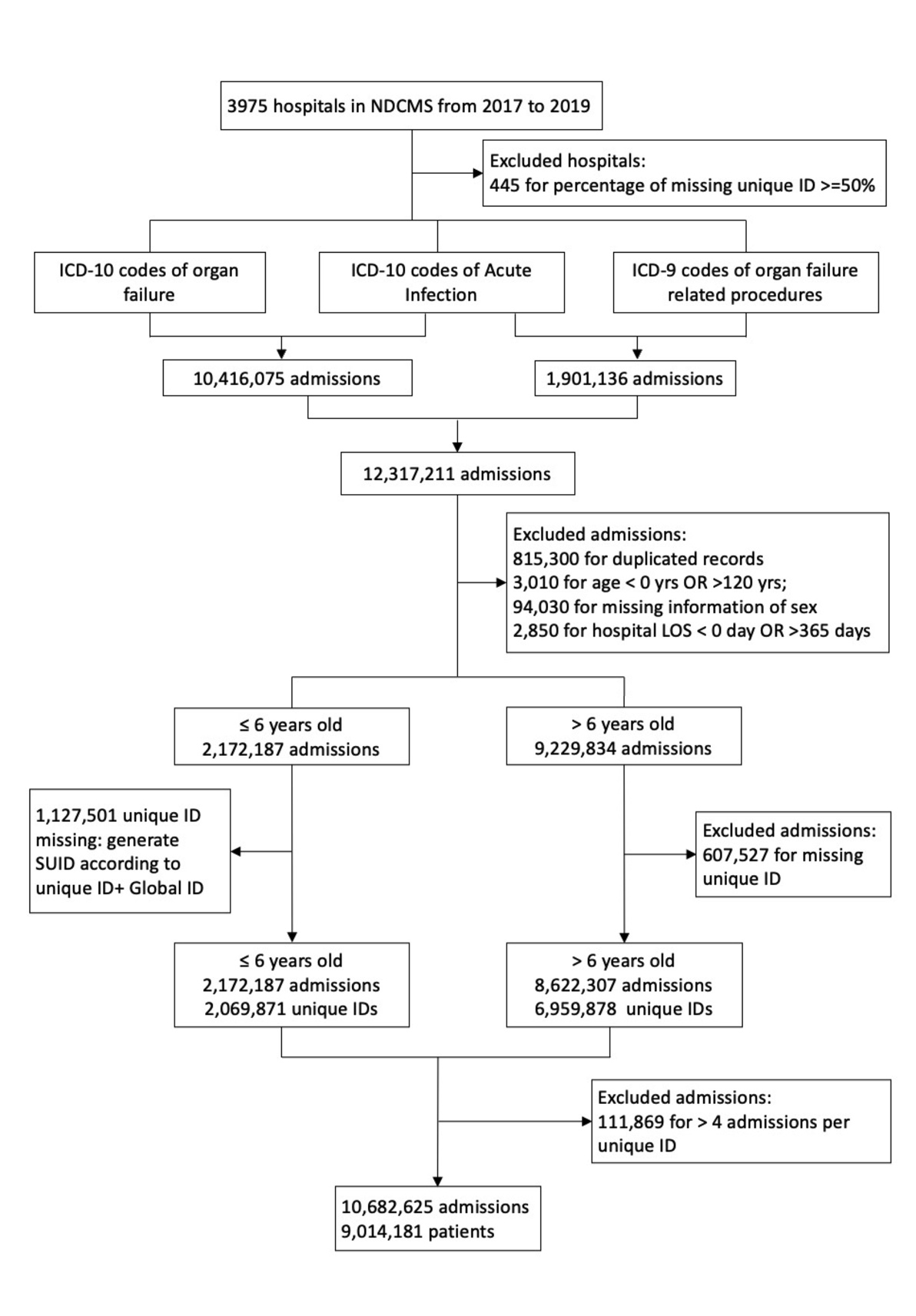
Figure S2. Enrollment of Admissions in NDCMS

A unique ID was created according to the 18-digit number of resident identity card in China for each patient. A unique global ID was created randomly in NDCMS for each hospital admission.

NDCMS= National Data Center for Medical Service; SUID= Structured Unique ID; yrs=years; LOS= Length of stay
